# Broad-scale evaluations of biodiversity change need reference comparisons

**DOI:** 10.1126/sciadv.aei1959

**Published:** 2026-10-28

**Authors:** James S. Sinclair, Daniela Cortés-Guzmán, Jukka Aroviita, Nathan J. Baker, Libuše Barešová, Catherine Bradley, Miguel Cañedo-Argüelles, David Cunillera-Montcusí, Hugh B. Feeley, Martial Ferréol, Mathieu Floury, Marie Anne Eurie Forio, Peter Goethals, Alexia M. González-Ferreras, Kaisa-Leena Huttunen, Ioannis Karaouzas, Aitor Larrañaga, S. Jannicke Moe, Ariane Moulinec, Diana Osadčaja, Petr Pařil, Andrés Peredo Arce, Marek Polášek, Michal Straka, Rudy Vannevel, Martin Wilkes, Peter Haase

**Affiliations:** ^1^ Senckenberg–Leibniz Institution for Biodiversity and Earth System Research, Senckenberg Research Institute and Natural History Museum Frankfurt, Gelnhausen 63571, Germany.; ^2^ Marine and Freshwater Solutions, Finnish Environment Institute, Oulu 90014, Finland.; ^3^ State Scientific Research Institute Nature Research Centre, Vilnius 08412, Lithuania.; ^4^ Czech Hydrometeorological Institute, Praha 143 00, Czech Republic.; ^5^ Environmental Protection Agency, Dublin D14 YR62, Ireland.; ^6^ SHE2 group (Surface Hydrology, Ecology and Erosion), FEHM-Lab, Institute of Environmental Assessment and Water Research, IDAEA, CSIC, Barcelona, Catalonia 08034, Spain.; ^7^ IFREMER–DYNECO/LEBCO, Centre de Bretagne, Plouzané, France.; ^8^ INRAE, UMR Agroecologie, Centre de Dijon, Dijon, Cedex 21065, France.; ^9^ INRAE, UR RiverLy, Centre de Lyon-Villeurbanne, Villeurbanne, Cedex, France.; ^10^ Department of Animal Sciences and Aquatic Ecology, Ghent University, Gent 9000, Belgium.; ^11^ IHCantabria—Instituto de Hidráulica Ambiental de la Universidad de Cantabria, C/Isabel Torres 15, Santander 39011, Spain.; ^12^ Ecology and Genetics Research Unit, University of Oulu, Oulu 90014, Finland.; ^13^ Institute of Marine Biological Resources and Inland Waters, Hellenic Centre for Marine Research, Anavyssos 19013, Greece.; ^14^ Department of Plant Biology and Ecology, University of the Basque Country (UPV/EHU), Leioa, Bilbao 48940, Spain.; ^15^ Norwegian Institute for Water Research (NIVA), Oslo 0579, Norway.; ^16^ Faculty of Biology, University of Duisburg-Essen, Essen 45141, Germany.; ^17^ Research Center One Health Ruhr, University Alliance Ruhr, Essen 45141, Germany.; ^18^ Department of Botany and Zoology, Faculty of Science, Masaryk University, Brno 611 37, Czech Republic.; ^19^ T. G. Masaryk Water Research Institute, p. r. i., 160 00 Praha, Czech Republic.; ^20^ Flanders Environment Agency, Aalst 9300, Belgium.; ^21^ School of Life Sciences, University of Essex, Colchester CO4 3SQ, UK.

## Abstract

Assessments of broad-scale biodiversity change commonly aggregate communities that differ in their initial impacts and their total degree of change (differing “impact trajectories”). Biodiversity responses may depend on the impact trajectory of a given community; thus, not controlling for these differences could produce a misleading mixture of trends even if all communities degraded or recovered. To evaluate this dependency, we related biodiversity trends for 4062 river invertebrate time series (1971 to 2024) from 23 European countries to impact trajectories for each community, quantified through comparison to least-impacted references. Trends were typically stronger and more consistent in initially more impacted communities that also exhibited more extreme total change. However, responses were weaker when initial impacts were lower and highly inconsistent with lesser total change. Our results show that biodiversity trends should be compared among communities with similar impacts, determined relative to appropriate references. Improving trend comparability is essential to accurately determine how biodiversity generally responds to anthropogenic impacts and conservation actions.

## INTRODUCTION

Broad-scale and long-term investigations of biodiversity change are more important than ever before. Such studies are crucial for guiding international efforts to halt biodiversity loss in terrestrial, marine, and freshwater ecosystems [e.g., Target 1 of the Kunming-Montreal Global Biodiversity Framework (KM GBF); (*1*)], for identifying the principal drivers of these losses (*2*, *3*), and for evaluating the effectiveness of conservation actions (*4*, *5*). However, biodiversity responses to anthropogenic impacts can be notoriously context dependent (*6*), varying even among communities exposed to the same stressors. This variability can arise for numerous reasons, including differences among communities in demography [e.g., stochastic variation in births and deaths; (*7*)], habitat conditions (*8*), species interactions (*9*), and connectivity to sources of new colonists (*10*). Broad-scale biodiversity studies often ignore these contextual differences by simply aggregating trends across numerous sites, which may obscure ecologically meaningful patterns, including evidence of general biodiversity loss that may appear as a heterogeneous mixture of trends (*11*–*14*). These points underscore the potential importance of local context for developing the accurate, broad-scale understanding of biodiversity change needed to determine how communities are responding to anthropogenic impacts and conservation actions.

One commonly missing piece of local context for biodiversity change, and thus a principal source of uncontrolled trend variability, is how each community changed in comparison to an unimpacted or least-impacted reference state (*15*, *16*). Instead, many studies just use the earliest years of each time series as the benchmark for change, which are not true references because these earlier years do not necessarily reflect preimpact conditions. Biodiversity research that lacks true references cannot determine: (i) how impacted each community was at the start of its time series; and (ii) how impacts changed over time relative to that starting point. Together, we refer to both aspects as each community’s “impact trajectory.” Communities with different impact trajectories, such as degrading communities that started at different initial levels of degradation, may have incomparable biodiversity trends because responses may vary along a continuous gradient of degradation or recovery (*17*). Continuing with degrading communities as our example, rare sensitive taxa may only be present in time series that began when the community was close to reference conditions and were then lost as the community degraded because of their high vulnerability to anthropogenic impacts (*18*, *19*). These losses would translate to strong declines in community metrics that are sensitive to changes in rare taxa, such as richness. In contrast, community time series that begin in a more impacted state may exhibit little to no response in richness as they further degrade because sensitive, rare taxa were already lost before the start of monitoring (*20*). Richness may even increase if new tolerant species, including non-natives, replace lost sensitive species (*21*, *22*). Therefore, a heterogeneous mixture of negative, neutral, and positive biodiversity trends could arise, even if all communities are degrading, by comparing communities with different trajectories. Similarly mixed responses could occur for recovering communities, and for any community metric, such as compositional metrics, which may remain stable and then shift rapidly only once a community reaches a certain threshold level of impact (*23*).

While biodiversity trends may vary depending on each community’s impact trajectory [see (*15*–*17*, *20*)], we still need empirical evidence demonstrating this occurs across broad spatial scales. Such evidence requires appropriate references and robust, temporal biodiversity data, which is commonly lacking because of the overall scarcity of consistent long-term monitoring data (*5*, *24*). As a result, we generally lack historical, preimpact data that could serve as a reference, and we lack biodiversity data with sufficient temporal resolution to quantify robust trends. Biomonitoring data, specifically for European river invertebrates, provides a feasible solution to both problems. River invertebrates have been collected by European biomonitoring programs for decades because they are important indicators of freshwater biodiversity and ecosystem health (*25*). In addition, as required by the European Union (EU) Water Framework Directive [see Annex V, Section 1.4 in (*26*)], each European country must identify stream-type specific, least-impacted reference communities for benchmarking the degree of degradation or recovery in sampled communities, i.e., using other communities instead of earlier years as the reference. These references are considered “least-impacted” because truly unimpacted references no longer exist across much of Europe. European river invertebrates therefore provide robust, broad-scale, temporal biodiversity data, along with the associated references needed to define the impact trajectory for each community.

Here, we quantified trends in taxon richness, total abundance, functional diversity, and taxonomic/trait composition for 4062 river invertebrate communities sampled across 23 European countries between 1971 and 2024 (Fig. 1). We examined the relationship between these trends and how communities changed in comparison to established references in each region. We expected that biodiversity trends would depend on the initial degree of impact in each community as determined by comparisons to reference communities, and on the total degree of community degradation or recovery (i.e., their impact trajectory). Our findings provide insight into the importance of reference comparisons in broad-scale biodiversity research, which helps guide efforts to evaluate global biodiversity change in terrestrial, marine, and freshwater ecosystems.

**Fig. 1.**
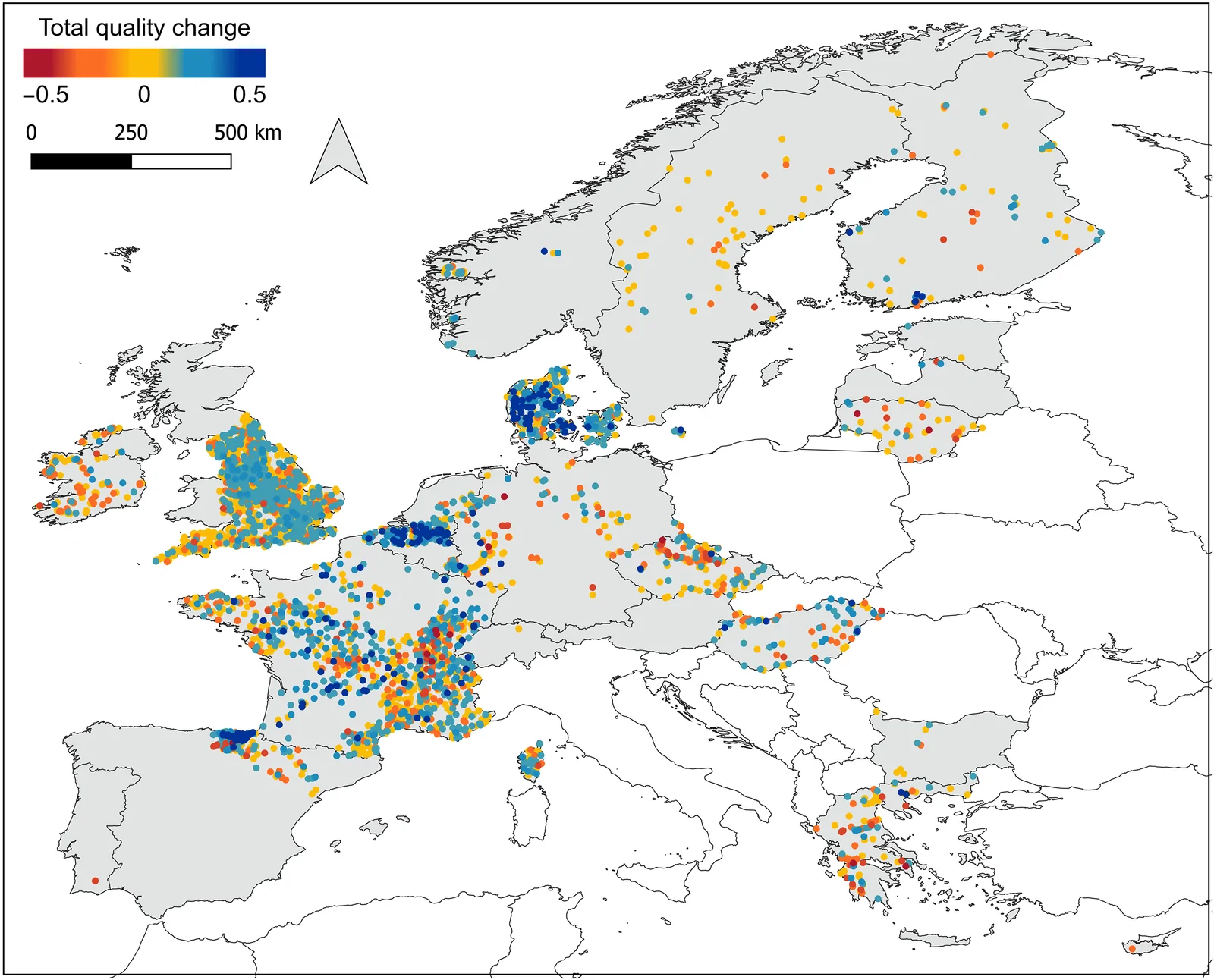
Locations of the 4062 European river invertebrate communities. Point colors indicate the total degree of “ecological quality” degradation or recovery, determined using temporal models of ecological quality described in our Materials and Methods and shown in Fig. 2. Stronger red colors indicate communities that degraded more through time and stronger blues indicate greater recovery.

## RESULTS

We quantified the initial degree of impact in each community and how impacts changed through time, based on temporal changes in “ecological quality” [calculated as the ecological quality ratio (EQR); see Materials and Methods]. EQRs are a biomonitoring index that uses selected taxa to indicate ecosystem quality, which has been applied throughout Europe since the implementation of the EU Water Framework Directive in 2000 (*26*). EQRs are commonly calculated on the basis of composition and/or diversity metrics for selected taxa (typically those sensitive or tolerant to pollution) or across all taxa. These metrics are converted to an observed:expected quotient through division by the metric values from least-impacted reference communities for the same river type in each country. The resulting quotient is the EQR, which typically ranges from 0 to 1. Initially poorer-quality communities are indicated by time series with EQR quotients that start closer to 0, which reflects observed values that are much lower than expected reference values, thus indicating higher initial impacts. Initially better-quality communities have higher starting EQR quotients closer to 1, which reflects lower initial impacts. EQRs that decline through time (i.e., negative total change) indicate a degrading community, and EQR increases (i.e., positive total change) reflect recovery. EQRs can therefore be used to quantify both the initial degree of impact in each community and how much each degraded or recovered through time, in a manner assumed to be comparable among countries (*27*).

### Ecological quality

On the basis of generalized additive mixed models (GAMMs) of EQR temporal changes, which can capture both linear and nonlinear changes (Fig. 2A), more sites recovered than degraded, with 2677 sites of 4062 total becoming more similar to their reference communities through time. Total changes ranged from maximum EQR declines of −0.65, reflecting substantial degradation compared to reference communities during a time series, to a maximum increase of +0.82. However, communities at the extreme ends of this range were rare, with the majority exhibiting much smaller total changes (e.g., 59% of sites only changed between −0.1 to +0.1 and 98% fell within −0.5 to +0.5). Therefore, to avoid predicting beyond the boundaries of our data in the following sections on biodiversity trends, we only predict how trends vary within a total EQR change range from −0.5 to +0.5. In addition, we use differing prediction ranges for the initial EQRs in degrading (from 0.5 to 1; ranging from half the reference value to equal to the reference value, respectively) versus recovering communities (from 0 to 0.5). We do so because total EQR changes of −0.5 to +0.5 are only realistic within certain initial EQR ranges. For example, a degrading community with an initial EQR of 1 can degrade by −0.5 through time, but a community with an initial EQR of 0.2 cannot because communities cannot decline below an observed:expected quotient of 0. Therefore, the prediction ranges must be adjusted to reflect plausible changes for each trajectory and so are not directly comparable between degrading and recovering communities, although they can be compared as a gradient from initially poorer to better quality and so are presented as such.

**Fig. 2.**
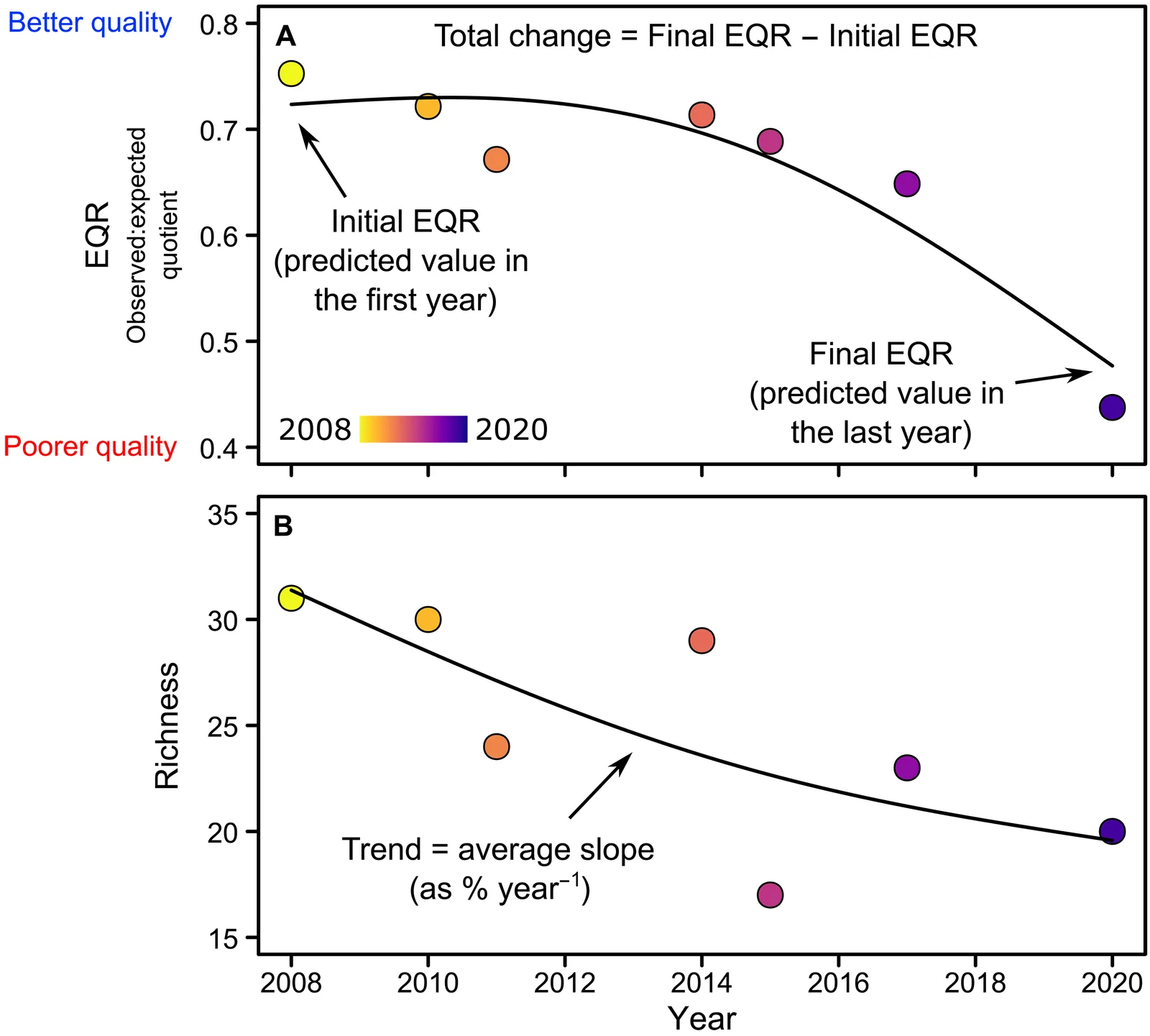
Temporal changes in ecological quality and biodiversity. Examples showing how we quantified: (**A**) initial ecological quality and total quality change using the EQR; and (**B**) trends in biodiversity metrics (using richness as an example) based on temporal GAMMs. Higher to lower initial EQRs respectively reflect communities whose impact trajectories began in an initially better to poorer state, with negative total quality change indicating degradation and positive change indicating recovery.

### Richness

Degrading communities generally lost taxon richness through time. The highest losses occurred in communities that started at an initially poorer ecological quality and that exhibited greater total degradation (i.e., those with lower initial EQRs and greater total EQR decline), which was evidenced by a significant initial EQR*total change interaction from GAMMs (Wald test, *n* = 4062, *F* = 132.9, edf = 10.9, *P* < 0.001; see table S1 for all GAMM results). For example, at a high total EQR degradation of −0.5 (left side of Fig. 3A), communities with a poorer initial EQR of 0.5 (dark purple line in Fig. 3A) had predicted richness losses of −4.5% year^−1^. At the same level of EQR degradation, communities with a better initial EQR of 1 exhibited lower predicted richness losses of −3.5% year^−1^ (yellow line in the left side of Fig. 3A). Note that trend differences of even 1% year^−1^ are substantial as annual changes accumulate over time, resulting in substantial losses or gains over decades, and the total estimated annual biodiversity change for a given taxonomic group or region often falls within just 0 to 2% year^−1^ (*13*, *22*, *28*). Predicted richness losses, and differences between initially poorer- and better-quality communities, became negligible at lower levels of total degradation, indicating highly variable trends across communities (see figs. S1 to S3 for the point distributions in all figures). Richness losses even switched to slight richness gains at the lowest degradation values (e.g., up to +0.4% year^−1^ as EQR decline approached 0 toward the center of Fig. 3A).

**Fig. 3.**
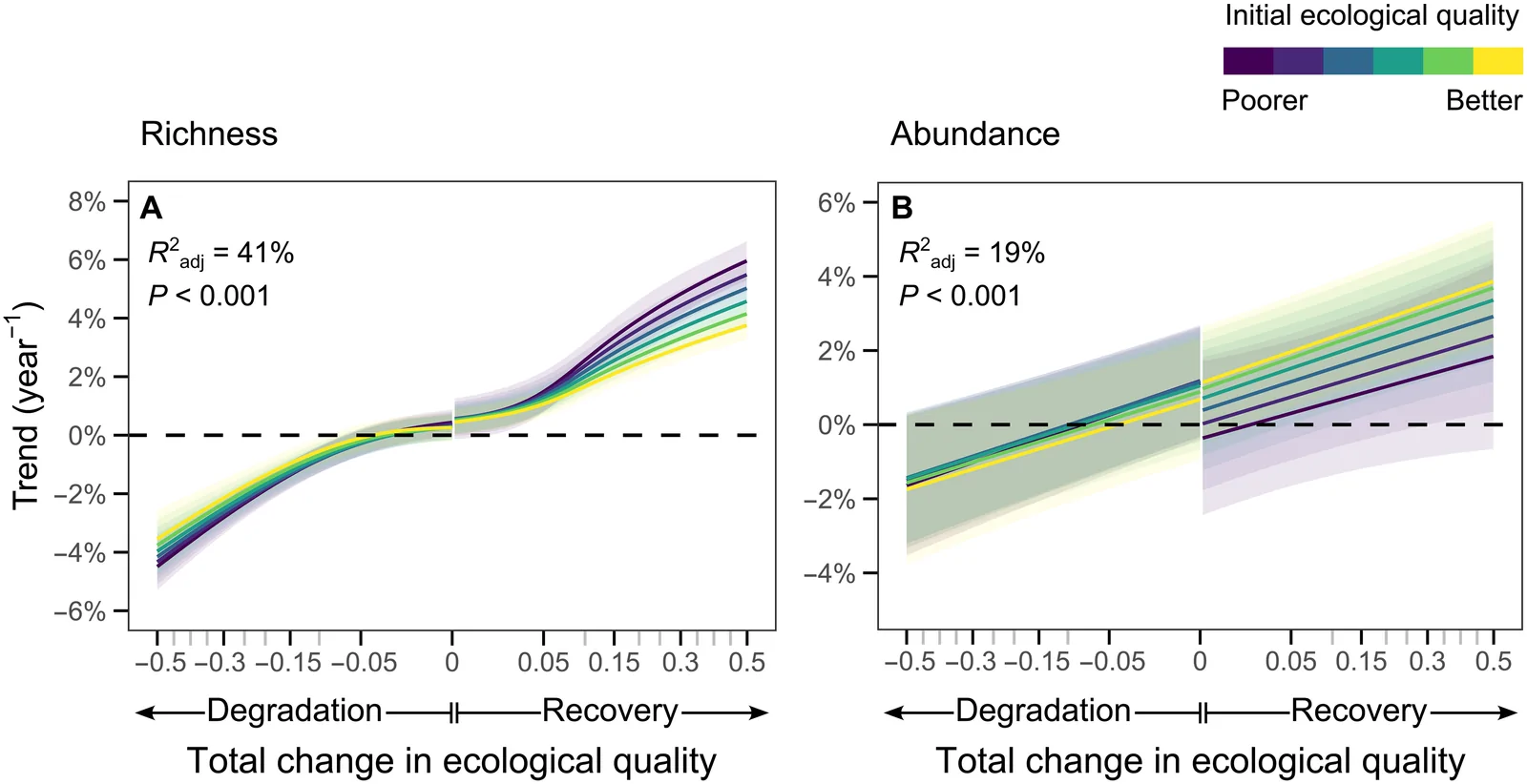
Richness and abundance. Temporal trends in (**A**) richness and (**B**) abundance (% year^−1^) in relation to initial ecological quality and total quality change, as determined from the EQR. Total quality change is shown on the *x* axis. Colored lines represent predicted initial ecological quality values. Note the ranges for the predicted initial ecological qualities differ between degrading (0.5 to 1, initially poorer to better quality respectively) and recovering communities (0 to 0.5). Predicted relationships were based on the associated GAMMs (*R*^2^_adj_ = adjusted *R*^2^). Shaded areas represent 95% confidence intervals.

Recovering communities generally gained richness, with greater gains occurring in initially poorer-quality communities that exhibited greater total recovery. For example, at a high total EQR recovery of +0.5 (right side of Fig. 3A), communities with a poorer initial EQR of 0 (dark purple line in Fig. 3A) had predicted richness gains of +5.9% year^−1^. At the same level of EQR recovery, communities with a better initial EQR of 0.5 had lower predicted richness gains of +3.7% year^−1^ (right side of the yellow line in Fig. 3A).

In addition to taxon richness, we examined trends in rarefied richness and coverage-based richness to test for biases in taxon richness arising from the need to adjust sampling effort due to variation in total community abundance among sites and years (*29*, *30*). We found no substantial differences in the responses of these metrics (see fig. S4), indicating that richness responses were robust to differences in community size.

### Abundance

Degrading communities primarily lost abundance when the degree of degradation was strongest (i.e., higher total EQR declines) and initial quality was better (left side of Fig. 3B), although differences among initially poorer- to better-quality communities were relatively weak. Abundance losses then switched to abundance gains at lower total degradation (moving from the left toward the center of Fig. 3B). For example, abundance was predicted to be lost at a rate around −1.6% year^−1^ at higher total EQR declines of −0.5 (averaged across different initial EQRs from 0.5 to 1). In contrast, abundance was predicted to be gained at a rate around +0.3% year^−1^ at lower total EQR declines of just −0.05 (Fig. 3B).

Recovering communities generally gained abundance at greater total quality recovery (right side of Fig. 3B), which was the opposite pattern compared to degrading communities. In addition, recovering communities exhibited stronger differences among initially better to poorer-quality communities. Specifically, recovering communities of an initially better quality (e.g., initial EQR of 0.5 shown in the upper yellow line on the right of Fig. 3B) tended to always gain abundance regardless of the amount of total EQR recovery, with stronger gains compared with initially poorer-quality communities. Furthermore, initially poorer-quality recovering communities even tended to lose abundance at lower degrees of total recovery (e.g., negative trends around lower total EQR increases of +0.05 and at an initial EQR of 0 shown in the lower dark purple line on the right of Fig. 3B).

Weaker abundance trend differences specifically among initially better- to poorer-quality degrading communities could be due to the lower sample size of this group (degrading: 1369; recovering: 2677). The influence of this sample size difference may be exacerbated in abundance, compared with other metrics like richness, as abundance trends tend to be more variable (e.g., compare the narrower richness trend range to the wider abundance range in fig. S1). To test for this, we repeated the abundance analysis after randomly resampling to produce an equal number of sites in each group. Trend differences between initially poorer- to better-quality degrading communities became more evident (fig. S5), providing further support that this gradient is likely present and reflects valid ecological responses, despite appearing to be weakly defined in our main analysis.

### Functional diversity

Total functional diversity exhibited similar responses as richness, with overall losses in degrading communities and gains in recovering communities (Fig. 4A). These responses were also stronger in initially poorer-quality communities that experienced greater total quality changes. For instance, at a total EQR decline of −0.5, communities with an initially poorer EQR of 0.5 lost 7.8% of their functional diversity year^−1^, whereas communities with an initially better EQR of 1 only lost 6.6% year^−1^.

**Fig. 4.**
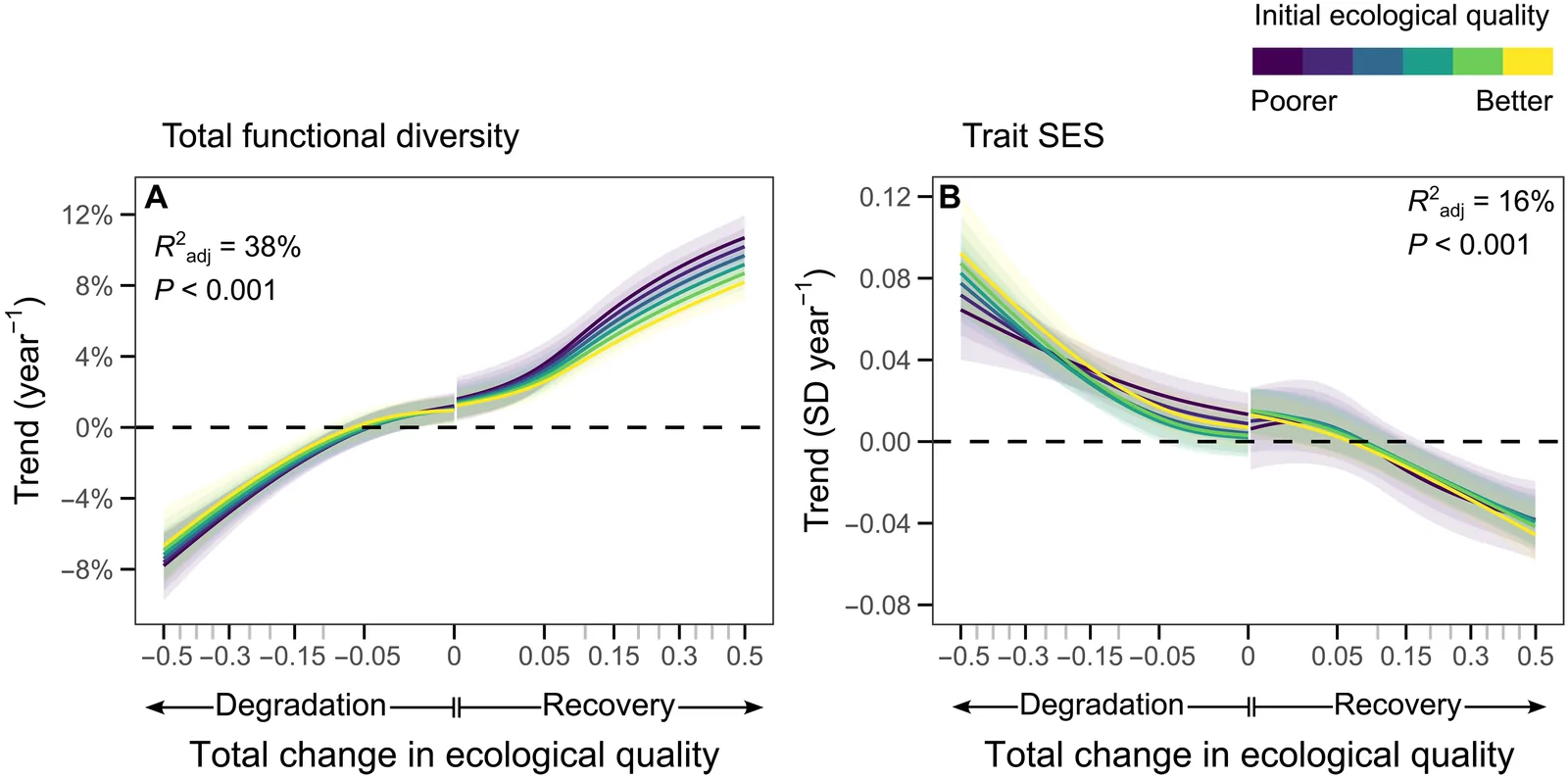
Functional diversity. Temporal trends in (**A**) total functional diversity (% year^−1^) and (**B**) trait SES (standardized effect size in units of SD per year; SD year^−1^) in relation to initial ecological quality and total quality change, as determined from the EQR. Total quality change is shown on the *x* axis. Colored lines represent predicted initial ecological quality values. Note the ranges for the predicted initial ecological qualities differ between degrading (0.5 to 1, initially poorer to better quality respectively) and recovering communities (0 to 0.5). Predicted relationships were based on the associated GAMMs (*R*^2^_adj_ = adjusted *R*^2^). Shaded areas represent 95% confidence intervals.

To help interpret how traits became distributed among taxa as both taxonomic and functional diversity changed, we also quantified trait standardized effect sizes (hereafter “trait SES”; see Materials and Methods). If trait SES declines through time, it indicates that the total functional distance among taxa became lower than expected for the given number of taxa in each community, which can occur when taxa cluster in trait space or generally share similar traits. If trait SES increases through time, it indicates that taxa are less clustered in trait space or are more functionally dissimilar. Our results showed that trait SES generally increased in degrading communities as they lost taxon richness and functional diversity through time (evidenced by overall positive predicted trait SES trends; Fig. 4B). This result indicates that functional diversity decreased less than expected as taxa were lost, meaning the remaining taxa still retained a broad range of trait values and so were generally functionally dissimilar. In contrast, trait SES declined in recovering communities as they gained taxa and functional diversity (evidenced by generally negative predicted trait SES trends; Fig. 4B), indicating that the gained taxa shared traits with already present taxa, resulting in lower than expected functional diversity and thus more functionally similar communities.

### Taxonomic and trait composition

Predicted temporal changes in taxonomic and trait composition always had confidence intervals that did not overlap 0, indicating that some compositional change occurred in all communities regardless of whether they degraded or recovered and regardless of how much total EQR change occurred (Fig. 5). Compositional changes always increased as total quality change increased, indicating greater compositional changes when communities experienced greater degradation or recovery. Both taxonomic and trait compositional changes were generally higher for initially poorer-quality communities. For example, degrading communities with a poorer initial EQR of 0.5 and a total EQR decline of −0.5 had predicted a taxonomic turnover of 0.11 SDs per year (SD year^−1^) or 1.1 after a decade (left dark purple line in Fig. 5A), potentially reflecting up to 50% or more turnover during a time series (*31*). In contrast, at a better initial EQR of 1, the same total degree of degradation resulted in a lower predicted turnover of 0.09 SD year^−1^ (left yellow line in Fig. 5A).

**Fig. 5.**
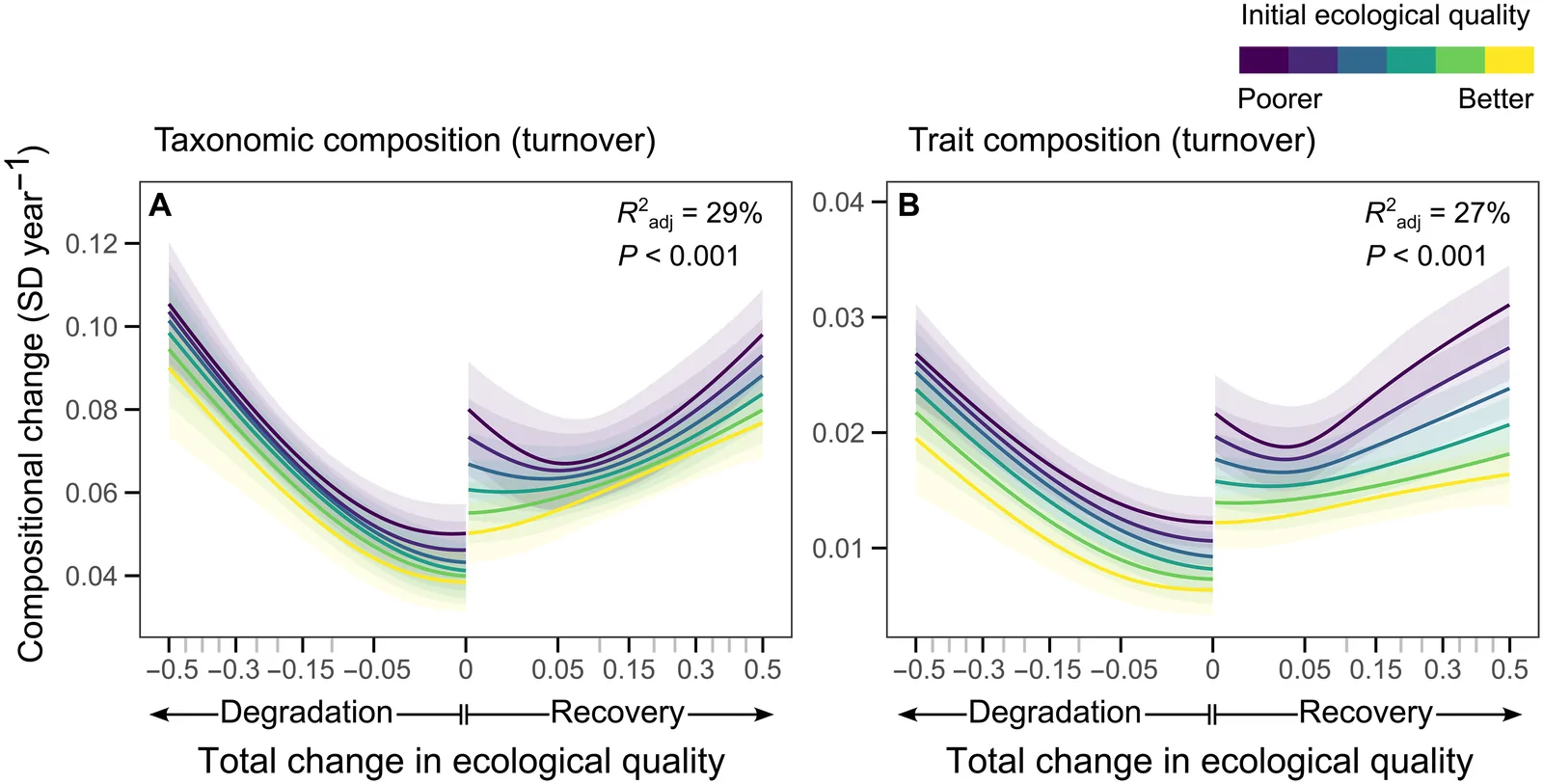
Taxonomic and trait composition. Temporal changes in (**A**) taxonomic and (**B**) trait composition (in units of SD per year; SD year^−1^) in relation to initial ecological quality and total quality change, as determined from the EQR. Total quality change is shown on the *x* axis. Colored lines represent predicted initial ecological quality values. Note the ranges for the predicted initial ecological qualities differ between degrading (0.5 to 1, initially poorer to better quality respectively) and recovering communities (0 to 0.5). Predicted relationships were based on the associated GAMMs (*R*^2^_adj_ = adjusted *R*^2^). Shaded areas represent 95% confidence intervals.

### Ecological quality and biodiversity

A caveat to our above results is that ecological quality is calculated using selected community measures, some of which overlap with the biodiversity metrics we analyzed (listed in table S2). Because multiple metrics are typically used, ecological quality can change even if some of its composite metrics do not and vice versa (e.g., see the wide range of responses shown in fig. S1), but this overlap still introduces the potential for circularity. To test for this influence, we reran our models including a factor indicating whether the biodiversity metric under consideration, or a component of it, was used in the ecological quality calculation for each site and compared the predicted relationships for sites without this overlap. We found the same general responses (fig. S6), excepting richness trends specifically in degrading communities, which were no longer stronger at initially poorer quality. These richness predictions, however, were based on only 33% of our total sites. Moreover, the corresponding patterns for the other biodiversity metrics remained generally unchanged, and functional diversity (which is not used for any ecological quality calculations) already supported the validity of the full richness results. We therefore retained the full dataset in our main analyses while presenting these additional results as sensitivity analyses.

## DISCUSSION

At more extreme levels of degradation or recovery, river invertebrate communities that were initially more impacted (i.e., poorer quality) at the start of their time series generally exhibited stronger biodiversity trends and greater compositional changes, compared with communities that were initially less impacted (i.e., better quality). These results can be explained if impacts—typically general environmental degradation and organic pollution (*32*)—in initially better-quality communities primarily affected only a subset of sensitive taxa, thus producing more muted overall community trends. In contrast, impacts in initially poorer-quality communities may have affected a broader variety of taxa, producing stronger trends (see an illustrated interpretation of these results in Fig. 6). Similar patterns have long been observed in polluted river invertebrate communities where biodiversity changes are minor at low-impact intensity because only certain sensitive taxa are lost and replaced with new, tolerant taxa. Then, community changes magnify once impacts intensify and exceed the tolerance thresholds of most species (*33*–*35*). Differing responses between initially poorer- versus better-quality communities becoming more pronounced with more extreme degradation or recovery likely occurred because biodiversity responses can be stronger and more consistent when examined across longer impact gradients (*20*, *36*, *37*) (Fig. 6).

**Fig. 6.**
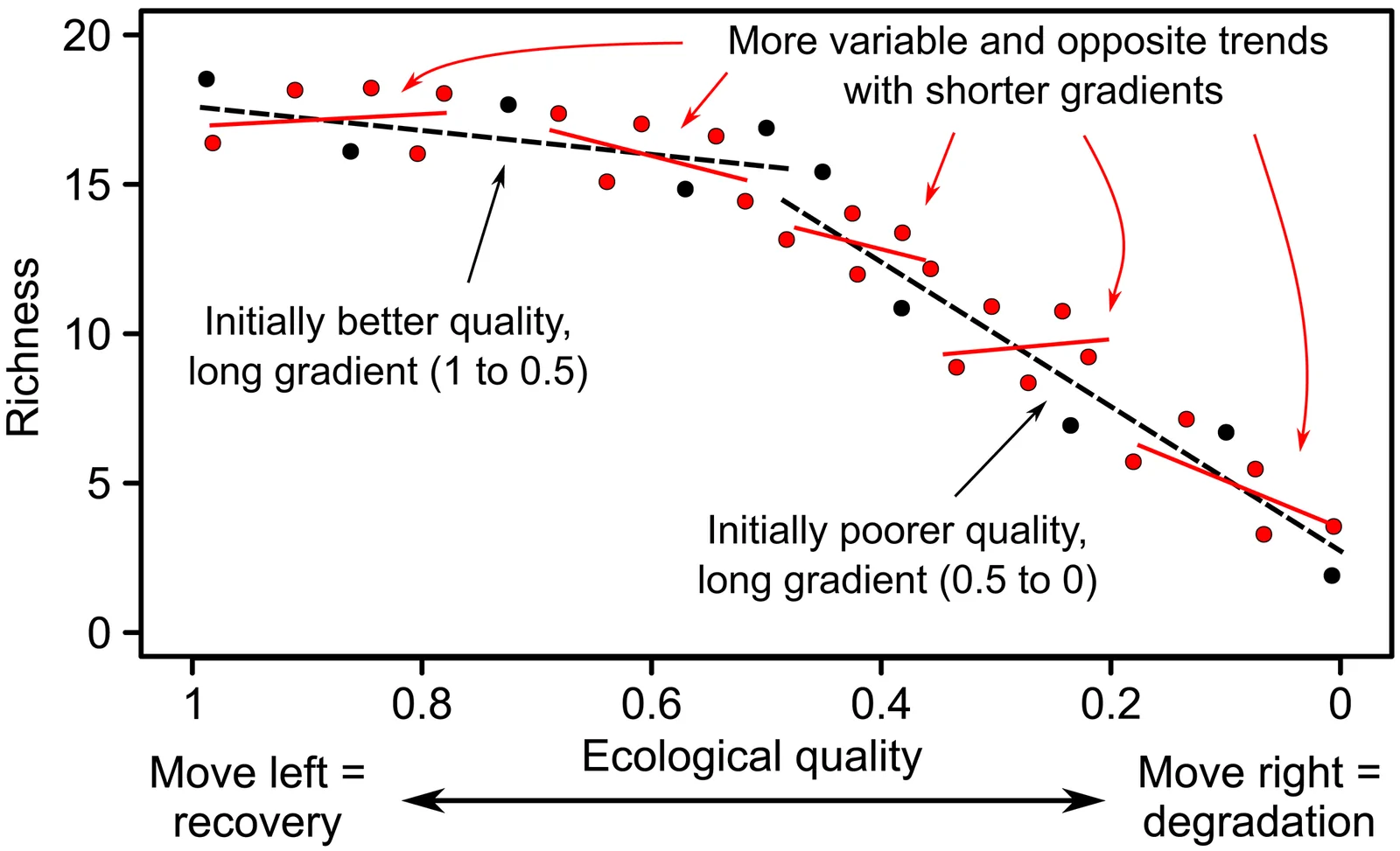
Conceptual illustration of community responses along differing impact trajectories. Example showing how river invertebrate communities can respond to stressor gradients [following responses to pollution from (*33*–*35*)] and how our results fit within this framework. In this example, richness generally declines as communities degrade (moving left to right in the figure) or increases as they recover (right to left). However, an initially better-quality community can exhibit a more muted trend (left black trend line, points indicate individual samples), despite experiencing large changes in ecological quality, because only a subset of sensitive taxa is lost and replaced by tolerant taxa as it degrades or vice versa as it recovers. In contrast, an initially poorer-quality community (right black line and points) can exhibit stronger trends as impacts are beyond the tolerance limits of most taxa. Communities sampled for an equivalent time but with shorter impact gradients (red points and lines) exhibit a more variable mixture of positive, neutral, and negative trends, which can produce overall opposite trends compared to communities with longer gradients and less difference between initially better- and poorer-quality sites.

However, the above patterns were more complex for particular biodiversity metrics, and for communities that experienced less extreme degradation or recovery, which encompassed the majority of sites. Specifically, abundance tended to exhibit stronger trends in initially better-quality communities, which was the opposite response compared with all other biodiversity metrics. This can be explained if abundance is primarily lost when degradation begins [e.g., common taxa decline first; (*38*, *39*)] or recovered last when impacts are addressed by conservation actions. Regarding sites that experienced lesser total change, these exhibited a more heterogeneous mixture of positive, negative, and neutral biodiversity trends, resulting in general trends that often overlapped zero, with little to no discernable differences between initially poorer- versus better-quality communities. This greater variability is not unexpected in communities with shorter impact gradients where biodiversity responses are likely to be more variable (*36*, *37*) (Fig. 6). The general trend could even be the opposite to those of communities that experienced greater quality changes, such as degrading communities gaining biodiversity or recovering communities losing it. Such counterintuitive community changes can occur when gains of tolerant taxa in degrading communities outweigh losses of sensitive taxa (*40*) or vice versa for recovering communities (*41*). Overall, our findings illustrate how variation among communities in their impact trajectories, i.e., their initial starting points and the lengths of their impact gradients, could produce a heterogeneous mixture of biodiversity trends, even if all communities have generally degraded or recovered. This commonly uncontrolled variation may partly explain the prevalence of mixed trends in broad-scale biodiversity research [see (*11*–*14*) for examples].

Greater biodiversity losses in initially poorer-quality, degrading communities further suggested that these types of communities may be the most vulnerable to future anthropogenic impacts. Not only did they lose a larger percentage of taxa, our results indicated they were then supported by a smaller number of functionally dissimilar and therefore more functionally unique taxa. These communities have been shown to be less resilient to additional impacts as further losses of the unique taxa that remain can trigger a collapse of both biodiversity and ecosystem functioning (*42*, *43*). This underscores the value of high biodiversity, and conservation actions to maintain and restore biodiversity, to better ensure that communities harbor greater redundancy and so are more resilient to future impacts.

Differing biodiversity changes between initially poorer- versus better-quality recovering communities also indicated how these community types may differ in their response to future conservation efforts, such as through the KM GBF (*1*) or the EU Nature Restoration Regulation (*44*). Biodiversity gains and compositional changes were generally higher in recovering communities of initially poorer quality, suggesting that severely damaged ecosystems may have the highest potential to recover rapidly in response to conservation actions (*45*). This potential is exemplified in actions across Europe during recent decades to address severe water pollution, which produced broad-scale biodiversity recovery (*46*, *47*), particularly in poorer-quality communities (*48*) during the 1990s and 2000s (*46*, *47*). In contrast, the weaker diversity and compositional changes in recovering and initially better-quality communities, and stronger abundance trends, likely occurred because, by this point, recovery was restricted to shifts between a limited subset of sensitive and tolerant taxa (Fig. 6) and abundance increases in a small number of common taxa. In addition, the later stages of recovery can be controlled by slower ecological processes, such as competition and dispersal (*49*), thus likely requiring longer timescales to detect whether recovery has occurred. Consequently, actions aiming for further recovery in better-quality communities may require adjusting the focal metrics used to gauge recovery, or adjusting expectations for monitoring time frames, and in some cases considering whether fully reaching reference conditions is feasible (*50*).

In addition to providing a comparative benchmark for community responses, reference comparisons helped identify which metrics may better serve to classify communities as degrading or recovering if broad-scale biodiversity studies lack reference data. Degrading communities generally lost richness and functional diversity through time, with the opposite response in recovering communities. Conversely, abundance exhibited a more inconsistent mixture of losses and gains, and both taxonomic and trait composition responded to degradation in a similar fashion as recovery, meaning that compositional change alone could not indicate whether communities underwent one process or the other. These results highlight that caution may be warranted when only using temporal trends in composite metrics of abundance [e.g., (*28*)] or composition [e.g., temporal ꞵ-diversity; (*12*, *13*)] to evaluate biodiversity change because, without reference comparisons, it may be difficult to conclude what trends in these metrics tell us about biodiversity status. If references are unavailable, then trait-based compositional metrics likely provide a more reliable option for determining degradation or recovery (*51*), particularly those like ecological quality that can reflect changes in both species and trait identity and shifts between known sensitive and tolerant taxa (*21*, *25*, *52*).

The greater consistency of responses in richness and functional diversity was somewhat unexpected because simple diversity metrics, particularly richness, are often considered unreliable indicators that depend on sampling effort, with responses that vary from one ecosystem to another (*51*, *53*, *54*). However, ours is not the only study that has found such consistent, broad-scale diversity responses to anthropogenic impacts (*2*, *3*, *22*). Whether simple diversity metrics provide inconsistent or consistent indicators likely depends on several factors. First, as shown here, the length of the impact gradient is undoubtedly important. Responses were highly variable in communities with short gradients and became more consistent as the total degree of impact increased [see (*22*) for similar findings], suggesting that studies mixing communities with differing impact gradient lengths may increase their chances of obtaining mixed trends. Second, the chosen study system may also affect response consistency. For instance, our results may show more consistent responses because European river invertebrate biomonitoring data provide an extremely large dataset, with samples generally collected using the same methods conducted at the same spatial scales across sites, all of which are key factors affecting response variability (*55*–*57*). In addition, common invertebrate collection methods (i.e., multihabitat samples) may reduce sampling effort biases by adjusting effort to the habitat heterogeneity of each site and targeting unique habitats harboring rare species (*58*). This conclusion is supported by the similar results that we found for rarefied and coverage-based richness. Trends in simple diversity metrics can therefore provide broadly reliable indicators of biodiversity responses to anthropogenic impacts, which can be used to track progress toward broad-scale conservation goals (e.g., Target 1 of the KM GBF to halt biodiversity loss). However, this indicator likely only works for systems with substantial impact gradients and when a sufficient quantity of data is collected following consistent and comprehensive methods. In the absence of suitable data quality, alternative indicators, such as the trait-based indices mentioned above and/or comparisons to reference conditions, are likely needed to obtain an accurate perspective of biodiversity change.

### Limitations and conclusions

Ecological quality is a widely collected European measure of integrated community responses to multiple freshwater stressors, which can be used to assess how waterbody health has changed through time (*59*), as an essential biodiversity variable (*60*), and to inform freshwater policy and legislation (e.g., the EU WFD; water.europa.eu/freshwater). Nonetheless, ecological quality is not calculated using the same references and methods in each region (listed in tables S2 and S3) and can only reflect changes in selected groups of sensitive and tolerant taxa. Therefore, although the degrees of quality change are broadly comparable among regions, the community components and stressors driving these changes can differ. The taxa involved may also not reflect responses to all potential stressors, such as salinization (*61*) or climate change (*62*). These limitations are, however, not exclusive to ecological quality as no single metric can perfectly reflect the full complexity of how biodiversity responds to all potential stressors in all regions. Furthermore, our analytical approach involved some degree of circularity given that we related biodiversity to ecological quality, meaning that we related one aspect of the community to another, thus predisposing the metrics to change as ecological quality changes. However, we generally found the same relationships even when this circularity was controlled (see fig. S6), and the wide response variability that we found across sites indicated that biodiversity could still respond independently of changes in ecological quality. Ultimately, the purpose of measures like ecological quality is not to diagnose exact drivers, nor to be entirely independent of other biodiversity metrics, but rather to summarize important community changes to steer conservation actions (*63*). In this sense, although ecological quality is not without its limitations, it still allows for broadly comparable evaluations of community responses to anthropogenic impacts in a manner that is informative for future management and policy.

An additional limitation is that the exact shape of our trend-trajectory relationships may be specific to our study system, i.e., European river invertebrate communities (and their references) sampled mostly during and after the 1990s, with trends primarily driven by changes in organic pollution and general environmental degradation. Further research is needed to examine these same relationships for other taxonomic groups, regions, or stressor types. For instance, some stressors can have strong, lethal impacts beyond a certain threshold [e.g., hypoxia from low dissolved oxygen; (*64*)]. This could produce trend-trajectory relationships that exhibit clearer thresholds than ours, with strong biodiversity trends in communities with impact trajectories that encompass the threshold and weak trends for those above and below it. Despite these potential differences, our study still provides empirical evidence of the general need for broad-scale biodiversity studies to consider and control for differing impact trajectories among communities, regardless of the nature of the trend-trajectory relationship in each system.

Our results show that considering and controlling the impact trajectory of each community in broad-scale biodiversity research likely provides key ecological context needed to better understand and compare biodiversity trends. This improved comparability is essential for uncovering consistent responses, reconciling diverging findings, and more accurately tracking broad-scale biodiversity loss and recovery to evaluate progress toward national and international conservation targets. The next challenge lies in determining how to better incorporate impact assessments via reference comparisons in future, broad-scale biodiversity research. Studies could compare communities to historical, predisturbance conditions at timescales relevant to the organisms in question, but such data likely do not exist in many global regions (*65*) and may not even provide realistic baselines (*50*). An alternative approach is to identify other less-impacted communities for comparison, similar to our use of spatial rather than temporal references. These approaches can be accomplished in a variety of ways, including meta-analyses of published experiments or surveys that compare impacted and control sites (*2*, *3*) or using environmental gradients to compare trends at lower versus higher impact levels (*66*), although this latter option can be hindered by a lack of long-term environmental data. Such approaches do not, however, come without drawbacks, including having to use potentially outdated references (*67*), needing to use modeled or highly altered conditions as the reference [e.g., for large rivers, (*68*); or agricultural ecosystems, (*69*)], or environmental changes also affecting reference sites (*70*). Regardless of the selected method, we encourage greater incorporation of reference comparisons in biodiversity research via the approaches outlined above, and advocate caution when interpreting weak or mixed trends as indicative of similarly weak or mixed outcomes of anthropogenic impacts and conservation actions.

## MATERIALS AND METHODS

### River invertebrate time series

We collated invertebrate time-series data for river sites across Europe from (*47*, *71*), from public repositories (e.g., environment.data.gov.uk), and from data provided by European freshwater researchers and managers. We defined the following site inclusion criteria: (i) abundance and community composition information was available for calculating different biodiversity metrics; (ii) ecological quality values could be calculated following methods compliant with the EU Water Framework Directive (detailed further below); (iii) at least seven individual years of data were available spanning at least a decade to enable robust trend estimation; (iv) all samples within a site were collected during the same season, defined as any three consecutive months; and (v) sampling methods were consistent within each site across years. These criteria led to the inclusion of data from 4062 sites. The included time series spanned years from 1971 to 2024. Most (>90%) began after the 1990s, with a mean total duration of 17.1 ± 5.4 years (mean ± SD) and an average of 11.5 ± 4.9 sampling years across time series (see table S3).

Most taxa in these data were identified to higher taxonomic levels, specifically 2848 out of 4062 sites had more than 75% of taxa identified at the family level or above. In some sites, identification levels were inconsistent through time; for these, we harmonized the level of identification by raising it to the highest recorded level, such as to family if identified to family level in some years and genus level in others. Higher-level identifications are common in invertebrate research where many taxa cannot be identified to the genus or species level and still preserve general community responses to environmental change (*72*). To confirm this, we repeated all analyses controlling for whether each community was identified at a “mixed” versus “family or higher” level and found no meaningful differences (see fig. S7), indicating that the coarser- and finer-level taxonomic data provided similar results. We therefore present the main results using all data.

Regarding the distribution of data among countries, the number of sites per country ranged from 1 to 1634 (mean ± SD of 176.6 ± 370.4). This uneven distribution may bias our results toward primarily reflecting responses in countries with larger sample sizes. To test for this, we reran our analyses using only data from 100 randomly selected sites for each country that provided data from more than 100 sites and repeated this process 1000 times. The averaged results across these iterations were consistent with our main results (see fig. S8), indicating that our findings are robust regardless of how much data came from each country. We therefore retained all sites in the dataset.

### Ecological quality

EQRs were calculated for each site and year following methods detailed for each region listed in table S2, given that the metrics and calculation methods involved differ among European regions. We calculated EQR temporal trends for each site on the basis of the modeled relationship between EQRs and their sampling years, performed using GAMMs conducted with the gamm function from the mgcv package in R (*73*, *74*). We used this approach because it allows for nonlinear responses (see Fig. 2A) and can control for temporal autocorrelation between successive years in the time series. All models included a smoothed, continuous term for year that generally used three basis dimensions (*k* = 3), although about 1% of sites required four or five dimensions. We chose this lower number of basis dimensions to allow for modeling linear or nonlinear responses depending on the time series, while avoiding overfitting in time series with the fewest data points. Models also included a first-order autoregressive structure with a continuous time covariate to control temporal autocorrelation in time series with uneven intervals between sampling years. We represented the initial state of each community as the predicted EQR in the first year and total change as the difference in the predicted EQRs between the first and last years (Fig. 2A).

### Biodiversity

We quantified biodiversity for each site and year based on six community metrics: (i) taxon richness, (ii) total abundance, (iii) taxonomic composition, (iv) trait composition, (v) total functional diversity, and (vi) trait standardized effect sizes. We examined taxonomic- and trait-based metrics to provide multiple perspectives of community change. Richness for each site and year was represented as the total number of taxa and abundance as the sum of all individuals in each community. Taxonomic composition for each site and year was represented as the Hellinger-transformed abundance of each taxon, with this transformation better ensuring that community differences are not solely determined by highly abundant taxa nor by shared taxon absences (*75*). In addition to taxon richness, we calculated rarefied richness using the rarefy function from the vegan package (*76*) and coverage-based richness using the iNEXT package (*77*) based on the expected number of species if 80% of each community was sampled.

Trait composition and total functional diversity were quantified using nine life-history and morphological traits (listed in table S4). Trait composition for each site and year was quantified as a “community-weighted mean” following the formula from (*78*)

$$\text{CWM}=\sum_{i=1}^{n}\;p_{i}\times {\text{trait}}_{i}$$

where CWM is the community-weighted mean for a given trait, $p_{i}$ is the Hellinger-transformed abundance of taxon *i* and ${\text{trait}}_{i}$ is the trait value for taxon *i*. Total functional diversity reflects the total functional distance among taxa and was calculated using the hill_func function from the hillR package (*79*) with a Hill number of *q* = 0.

Values for all traits were obtained from (*80*, *81*). Missing trait values for a given taxon were gap-filled either using values from a higher taxonomic level (e.g., the genus-level value for a species-level identification), or if this was missing, by averaging values across taxa within a higher-level group (e.g., using the average value across species within the genus). Using traits from higher-level taxonomic groupings to infer values for lower-level taxa is common in invertebrate research and still broadly maintains trait-environment relationships (*82*). To ensure that all traits contributed equally, we converted trait values to proportions, which allows for combining fuzzy-coded traits that use different value ranges, and we weighted each trait using weights obtained from a Multiple Factor Analysis [performed with the FactoMineR package; (*83*)], which ensures that traits composed of more modalities (shown in table S4) have equal weight to those composed of fewer.

We calculated trait standardized effect sizes by randomly shuffling trait values among taxa without replacement, thereby reassigning traits to taxa while maintaining trait structure, and repeating this 1000 times. For each permutation, we recalculated total functional diversity for each community to produce a null distribution of values for the given number of taxa expected by chance. We then subtracted the mean of the null distribution from observed total functional diversity and divided by the SD of the null distribution.

### Temporal biodiversity trends

Temporal changes in all metrics were calculated following the same modeling methods used for ecological quality. Given these relationships can be nonlinear, we quantified the overall trend as the average predicted slope (i.e., the first derivative) across all years in the time series, whether a year had data or not (Fig. 2B). Average slopes for richness, abundance, and total functional diversity were converted to percent change per year, which required log-transforming each metric before modeling, then exponentiating the average slope, subtracting 1, and multiplying by 100. This conversion ensured that trends were comparable among sites regardless of differences in abundance units, total diversity, or the level of taxonomic identification. Trait SES slopes were not converted to percent change because they are in units of SD, which are already comparable across communities.

Temporal changes in both taxonomic and trait composition were quantified using detrended correspondence analysis (DCA). We chose this approach because DCA can represent temporal compositional change in a similar manner as other alternative approaches, such as temporal β-diversity, but allows for retaining all data in each time series and does not require choosing a particular year for comparison. We first ordinated composition for all years in each site using DCA, performed using the decorana function from vegan. We then extracted the scores for each year on the first DCA axis, modeled these scores in relation to sampling year, and calculated the average slope following the same methods as the other community metrics (Fig. 2B). DCA scores are in units of SD of compositional turnover; thus, the slopes are in units of SD year^−1^. A change of 1 to 2 SDs represents about 50% compositional change, and 4 SDs represents about 100% (*31*).

### Statistical analyses

We related biodiversity trends to the initial ecological quality of each community, and the total change in ecological quality, using separate GAMMs for each metric. The response variable for each model was either the slopes of richness, abundance, or total functional diversity (% change year^−1^), or the slopes of trait SES or compositional change (SD year^−1^). The predictors for all models included an anisotropic smoothed interaction between initial ecological quality (untransformed) and total quality change (square-root transformed to reduce clustering at lower values). The smoothed interaction term used the default basis dimensions, and we checked model diagnostics, including the need for higher basis dimensions, using the gam.check function. In addition to the smoothed interaction, we included fixed continuous predictors for: (i) site latitude and longitude to control for broad-scale spatial variation in trend values; (ii) total time series duration to control for slope differences among shorter to longer time series; and (iii) the initial year of each time series to control for slope differences among time series spanning different time periods. These terms explained up to 7% of total deviance depending on the model. We also included random intercept terms for the country of each site to control for any differences among countries (e.g., sampling methods; table S3) and for drainage basin to control for nonindependence among hydrologically connected sites within the same basin. In addition, we checked for fine-scale spatial autocorrelation among trends based on Moran’s eigenvector maps [MEMs; (*84*)] for six separate Euclidean distance threshold neighborhood matrices whereby sites were considered connected if they were within either 1, 5, 10, 25, 50, or 100 km of one another. None of the resulting MEMs explained >1% of total trend variation at any distance threshold, so we considered fine-scale spatial autocorrelation as having little influence on our results and did not control for it in our models. All GAMMs used a Gaussian distribution (identity link). The significance (*P* < 0.05) of the smoothed interaction was determined using Wald-type tests (*73*).

## Supplementary Materials

### This PDF file includes:

Figs. S1 to S8 Tables S1 to S4 References
