# Supplementary material for: Broad-scale evaluations of biodiversity change need reference comparisons
Source: Sci Adv. 2026 Oct 28;12(44):eaei1959. doi: 10.1126/sciadv.aei1959 (PMC13715692; doi:10.1126/sciadv.aei1959)
Supplement: Supplementary file 1 [file sciadv.aei1959_sm.pdf]

Science Advances
AAAS

Supplementary Materials for

Broad-scale evaluations of biodiversity change need reference comparisons

James S. Sinclair *et al.*

Corresponding author: James S. Sinclair, james.sinclair270@gmail.com

Sci. Adv. **12**, eaei1959 (2026)
DOI: 10.1126/sciadv.aei1959

This PDF file includes:

Figs. S1 to S8
Tables S1 to S4
References

Fig. S1.

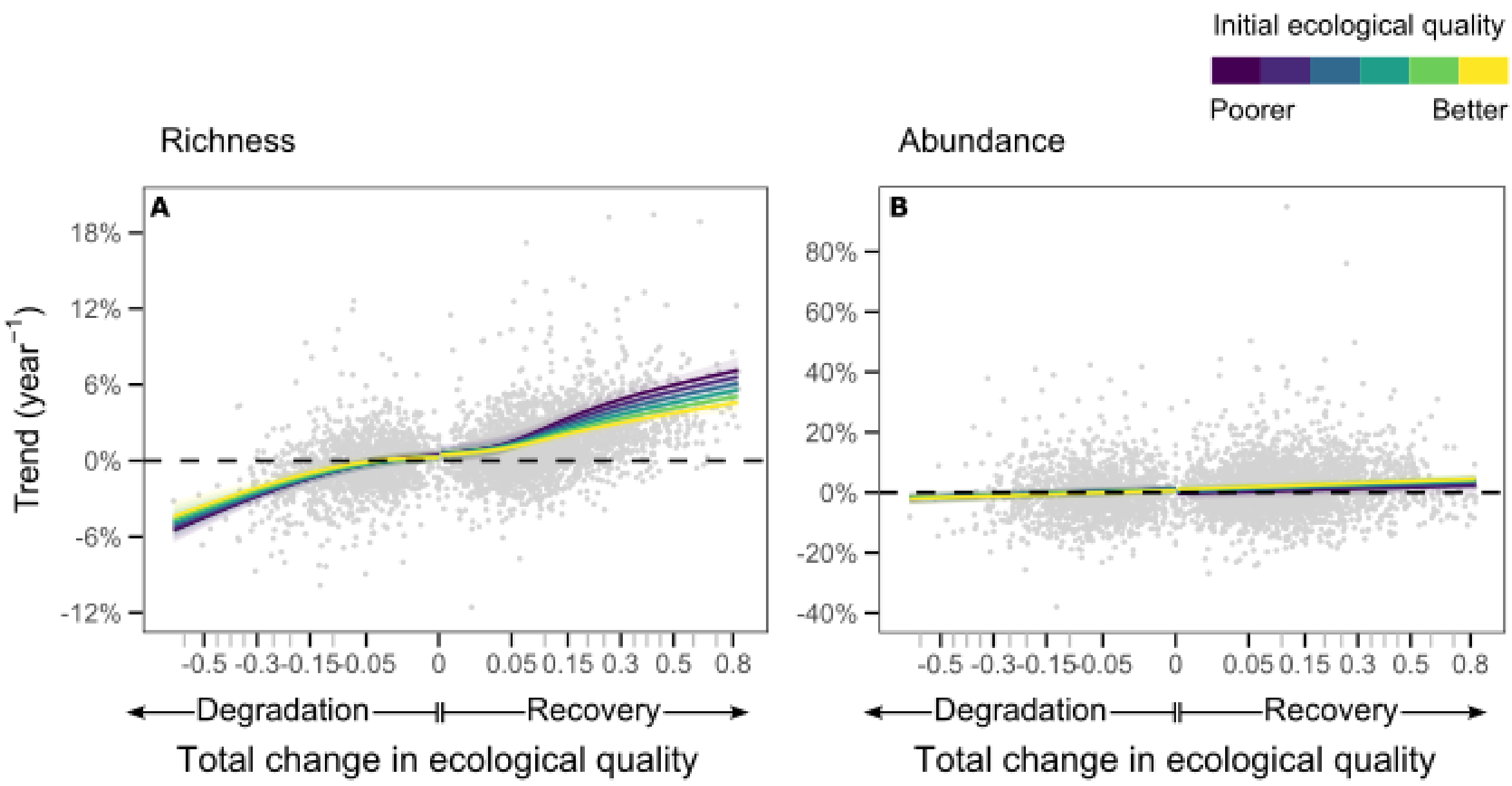

Data distribution and temporal trends in (A) richness and (B) abundance (% year^{-1}) in relation to initial ecological quality and total quality change, as determined from the Ecological Quality Ratio (EQR). Total quality change is shown on the x-axis. Gray points show raw observations, and colored lines represent predicted initial ecological quality values, which differ between degrading and recovering communities (degrading: range of 0.5–1 as initially poorer to better quality, respectively; recovering: 0–0.5). Predicted relationships were based on the associated generalized additive mixed models (GAMMs; R^2_{adj} = adjusted R^2). Shaded areas represent 95% confidence intervals.

Fig. S2.

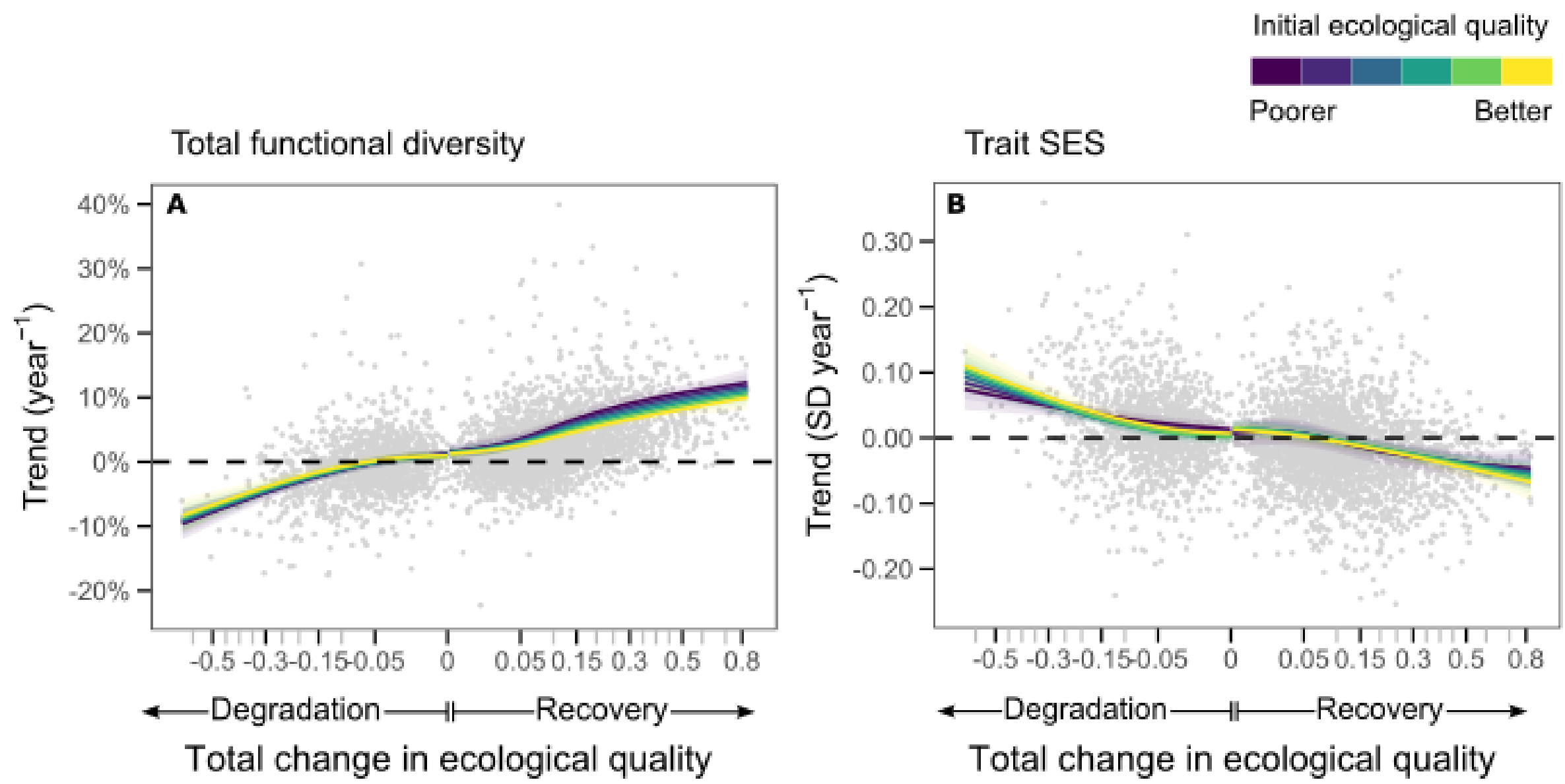

Data distribution and temporal trends in (A) total functional diversity (% year^{-1}) and (B) trait SES (SD year^{-1}) in relation to initial ecological quality and total quality change, as determined from the Ecological Quality Ratio (EQR). Total quality change is shown on the x-axis. Gray points show raw observations, and colored lines represent predicted initial ecological quality values, which differ between degrading and recovering communities (degrading: range of 0.5–1 as initially poorer to better quality, respectively; recovering: 0–0.5). Predicted relationships were based on the associated generalized additive mixed models (GAMMs; R^2_{adj} = adjusted R^2). Shaded areas represent 95% confidence intervals.

Fig. S3.

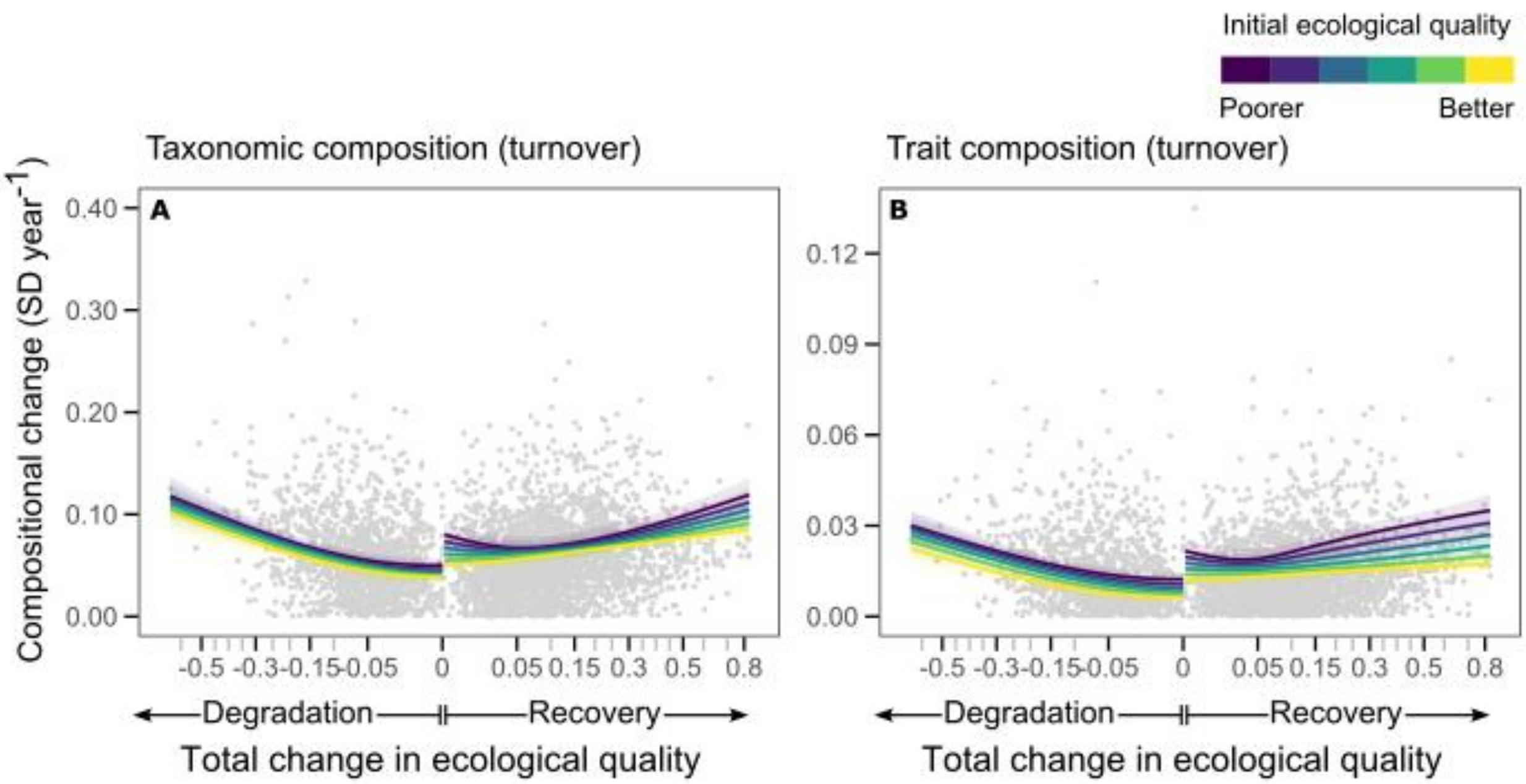

Data distribution and temporal changes in (A) taxonomic and (B) trait composition (SD year^{-1}) in relation to initial ecological quality and total quality change, as determined from the Ecological Quality Ratio (EQR). Total quality change is shown on the x-axis. Gray points show raw observations, and colored lines represent predicted initial ecological quality values, which differ between degrading and recovering communities (degrading: range of 0.5–1 as initially poorer to better quality, respectively; recovering: range of 0–0.5). Predicted relationships were based on the associated generalized additive mixed models (GAMMs; R^2_{adj} = adjusted R^2). Shaded areas represent 95% confidence intervals.

Fig. S4.

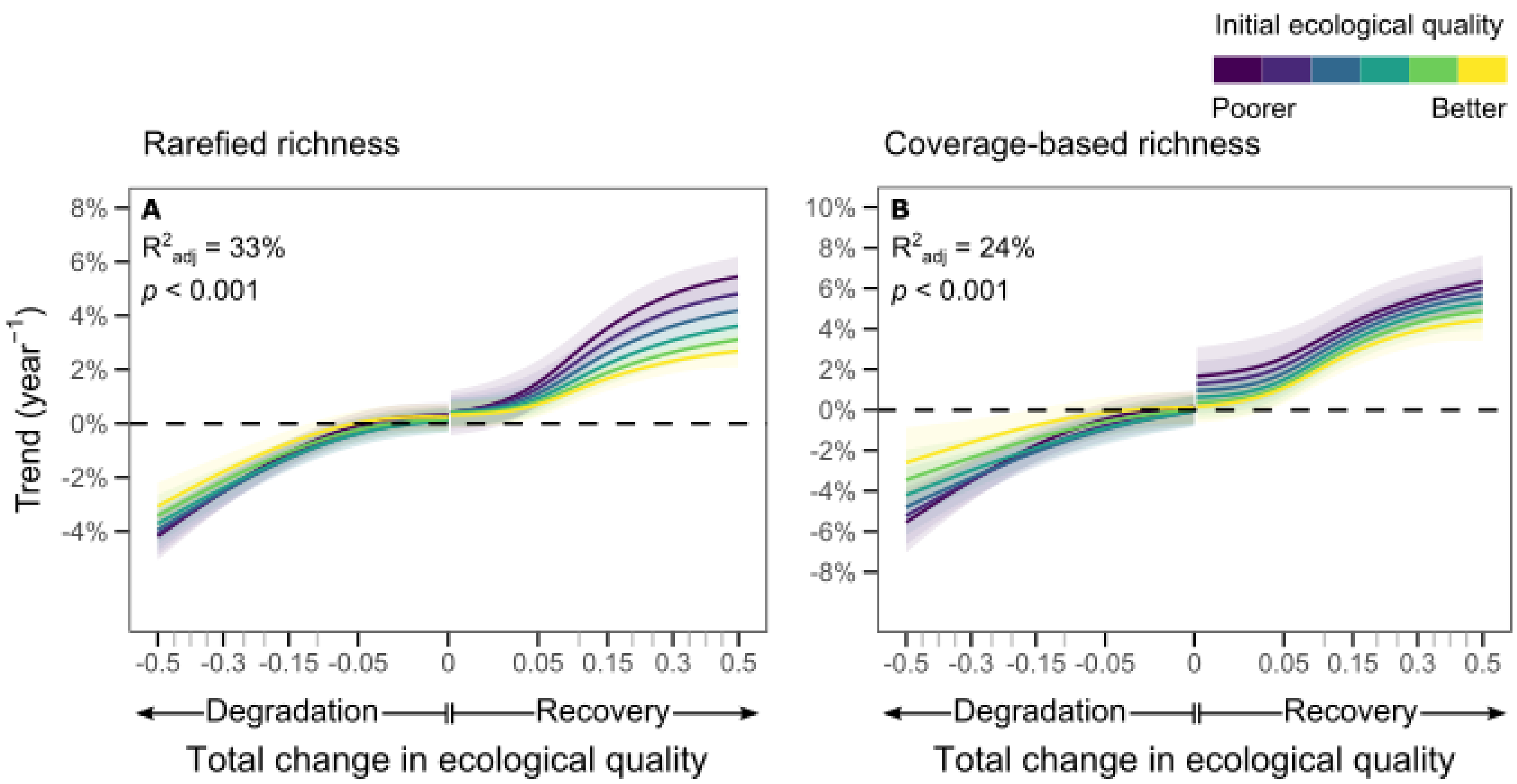

Temporal trends in (A) rarefied and (B) coverage-based richness (% year^{-1}) in relation to initial ecological quality and total quality change, as determined from changes in the Ecological Quality Ratio (EQR). Total quality change is shown on the x-axis, with colored lines representing predicted values for initially poorer to better ecological qualities (degrading: 0.5–1 as initially poorer to better quality, respectively; recovering 0–0.5), based on the associated generalized additive mixed models (GAMMs; R^2_{adj} = adjusted R^2). Shaded areas represent 95% confidence intervals.

Fig. S5.

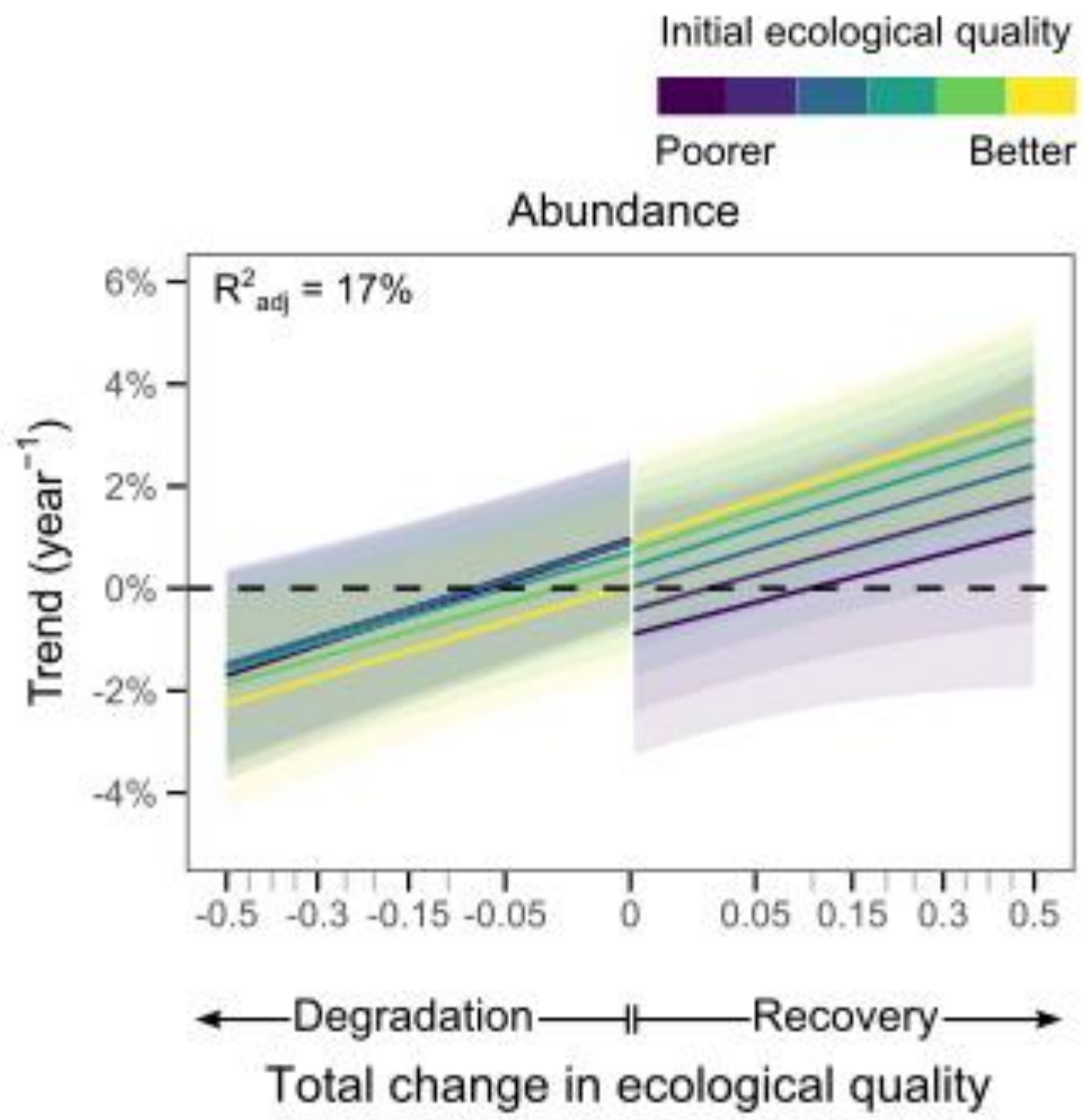

Temporal trends in abundance (% year-1) in relation to initial ecological quality and total quality change, as determined from changes in the Ecological Quality Ratio (EQR). To balance the sampling sizes between degrading and recovering communities, we randomly resampled recovering sites to match the number of degrading sites (n = 1,369) and repeated this 1,000 times. Predicted lines and adjusted R^2 (R^2_{adj}) reflect averaged values across iterations. Total quality change is shown on the x-axis, with colored lines representing predicted values for initially poorer to better ecological qualities (degrading: 0.5–1 as initially poorer to better quality, respectively; recovering 0–0.5), based on the associated generalized additive mixed models (GAMMs). Shaded areas represent 95% confidence intervals.

Fig. S6.

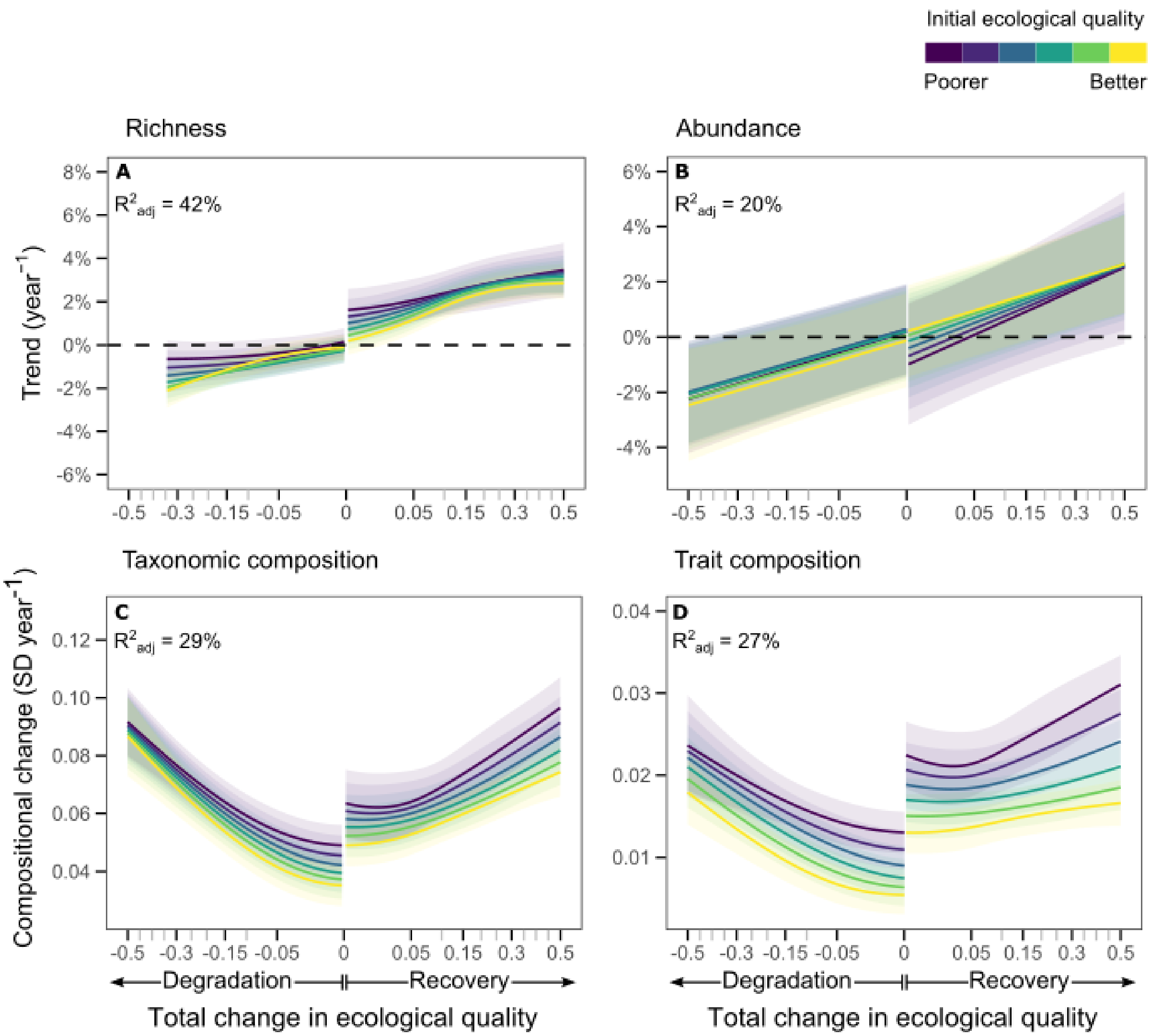

Temporal trends in (A) richness and (B) abundance (% year^{-1}), and (C) taxonomic and (D) trait composition (SD year^{-1}), in relation to initial ecological quality and total quality change, as determined from changes in the Ecological Quality Ratio (EQR). Predicted values are shown for the subset of sites where the corresponding biodiversity metric was not used to calculate the EQR, avoiding potential circularity (57% of sites for richness, 95% for abundance, 86% for taxonomic composition, and 74% for trait composition). Total quality change is shown on the x-axis, with colored lines representing predicted values for initially poorer to better ecological qualities (degrading: 0.5–1 as initially poorer to better quality, respectively; recovering 0–0.5). The x-axis ranges are adjusted to the observed data distribution, resulting in the exclusion of the most extreme values for degrading communities in the richness panel. Predictions are based on the associated generalized additive mixed models (GAMMs). Shaded areas represent 95% confidence intervals.

Fig. S7.

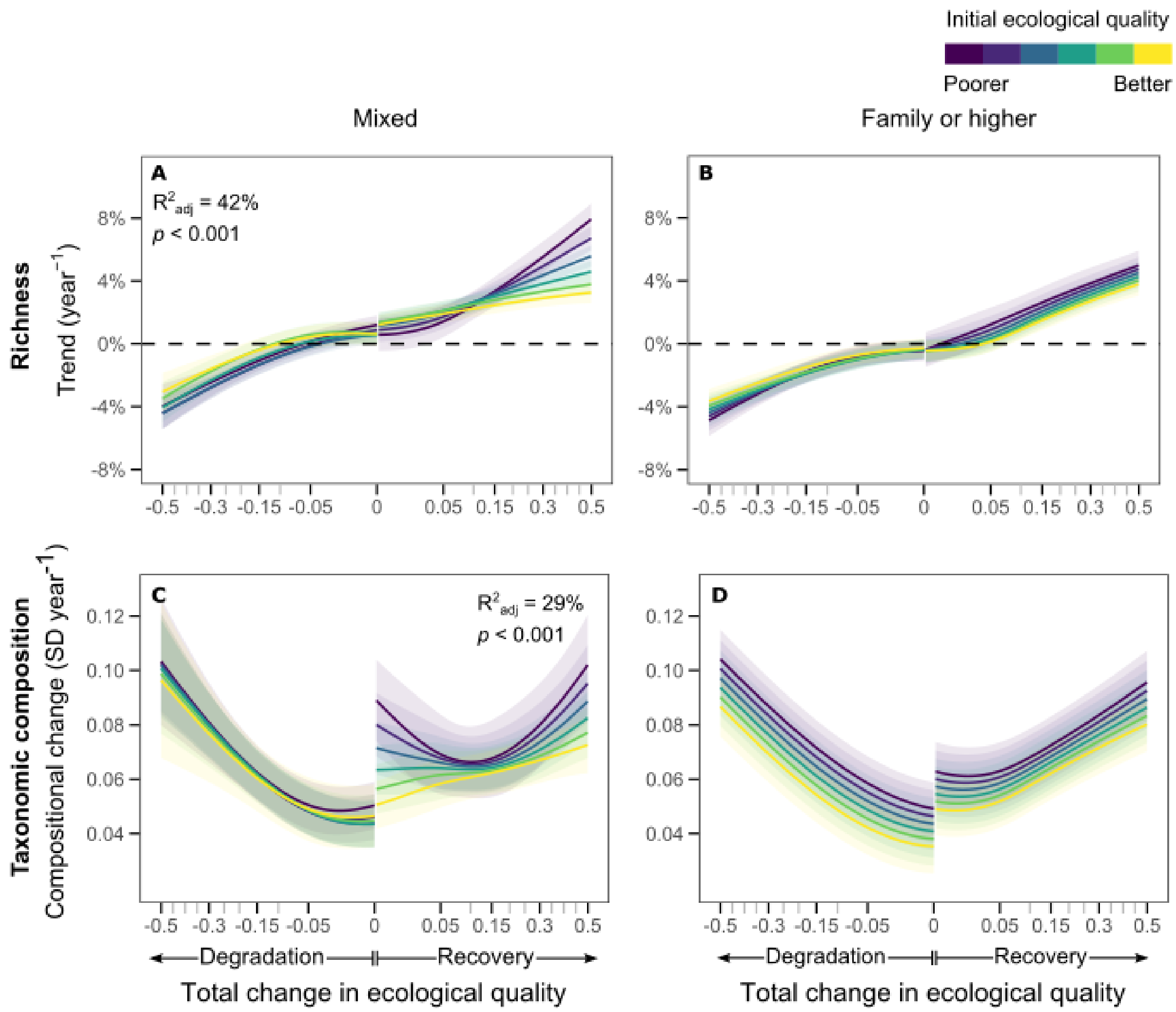

Temporal trends in (A, B) richness (% year^{-1}) and (C, D) taxon composition (SD year^{-1}) in relation to initial ecological quality and total quality change, as determined from changes in the Ecological Quality Ratio (EQR). Predicted values are shown separately for sites with mixed taxonomic resolution (A, C; 28% of sites combining family-, genus-, and species-level identifications) and for sites with coarser resolution (B, D; 72% of sites with ≥75% of taxa identified at family or higher level). Total quality change is shown on the x-axis, with colored lines representing predicted values for initially poorer to better ecological qualities (degrading: 0.5–1 as initially poorer to better quality, respectively; recovering 0–0.5), based on the associated generalized additive mixed models (GAMMs; R^2_{adj} = adjusted R^2). Shaded areas represent 95% confidence intervals.

Fig. S8.

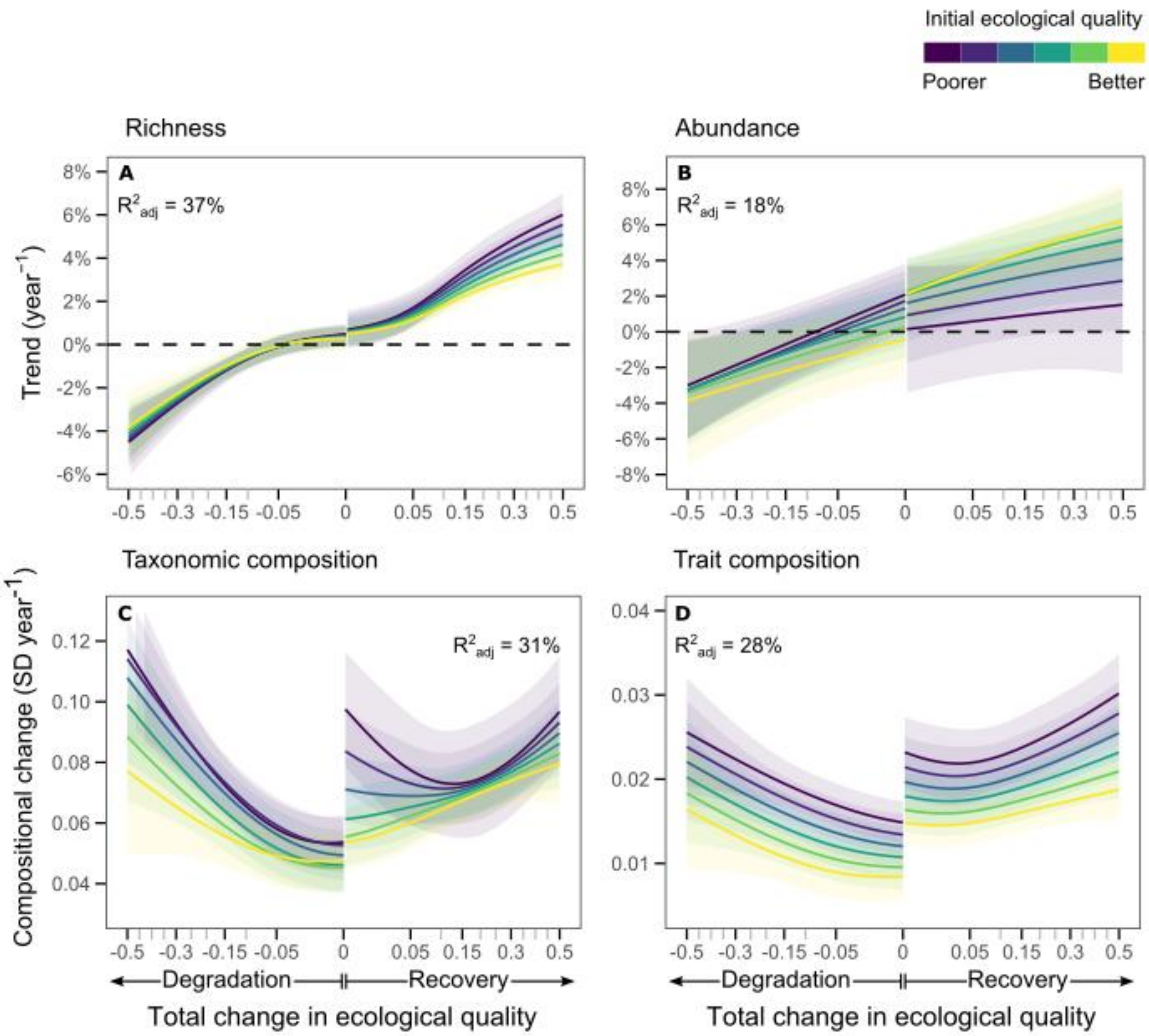

Temporal trends in (A) richness and (B) abundance (% year^{-1}), and (C) taxonomic and (D) trait composition (SD year^{-1}), in relation to initial ecological quality and total quality change, as determined from changes in the Ecological Quality Ratio (EQR). To balance sampling sizes across countries, we randomly resampled 100 sites from countries with more than 100 sites and repeated this 1,000 times. Predicted lines and adjusted R^2 (R^2_{adj}) reflect averaged values across iterations. Total quality change is shown on the x-axis, with colored lines representing predicted values for initially poorer to better ecological qualities (degrading: 0.5–1 as initially poorer to better quality, respectively; recovering 0–0.5), based on the associated generalized additive mixed models (GAMMs). Shaded areas represent 95% confidence intervals.

Table S1.

Response variable	*n*	*P*	R^2_{adj}	F	edf
Richness	4,062	<0.001	0.41	132.89	10.95
Abundance	4,062	<0.001	0.19	11.59	5.70
Total functional diversity	3,962	<0.001	0.38	146.76	9.89
Trait SES	3,962	<0.001	0.16	30.14	13.23
Taxon composition	4,062	<0.001	0.29	30.99	12.81
Trait composition	4,062	<0.001	0.27	37.29	13.00
Rarefied richness	4,062	<0.001	0.33	81.14	14.37
Coverage-based richness	4,062	<0.001	0.24	54.44	12.60
Richness–mixed ID	4,062 (1,214)	<0.001	0.42	42.28	13.29
Richness–family ID	4,062 (2,848)			82.87	12.30
Taxonomic composition–mixed ID	4,062 (1,214)	<0.001	0.29	11.00	11.23
Taxonomic composition–family ID	4,062 (2,848)			62.55	2.75
Richness–'without circularity'	4,062 (2,298)	<0.001	0.42	49.10	10.68
Richness–'with circularity'	4,062 (1,764)			97.46	12.26
Abundance–'without circularity'	4,062 (3,726)	<0.001	0.20	11.40	5.03
Abundance–'with circularity'	4,062 (336)			4.40	7.92
Taxonomic composition–'without circularity'	4,062 (3,475)	<0.001	0.29	32.05	8.99
Taxonomic composition–'with circularity'	4,062 (587)			10.61	11.12
Trait composition–'without circularity'	4,062 (2,995)	<0.001	0.27	38.46	10.65
Trait composition–'with circularity'	4,062 (1,067)			12.81	9.71

GAMM summary statistics. For each model, *n* indicates the number of observations, *P* and R^2_{adj} indicate the significance and overall model fit respectively, and F and edf correspond to the F-statistic and effective degrees of freedom of the interaction term between initial EQR and total change. Note the difference in *n* for the trait models as some communities had too few taxa to quantify functional diversity. For models including a grouping factor (taxonomic identification level or circularity), values in parentheses indicate the number of observations in each group.

Table S2.

Country	Method	Metrics	Method reference	Circularity
Austria	Multimetric index using three metrics	(1) Saprobic index; (2) % of oligosaprobic taxa; (3) Rhithron-type index; (4) Index of biocoenotic region; (5) Proportion of akal-lithal-psammal preferring taxa; (6) BMWP	(*85*)	Taxonomic composition
Belgium	Multimetric Macroinvertebrate Index Flanders	(1) Total taxon richness; (2) number of EPT (Ephemeroptera, Plecoptera and Trichoptera); (3) Shannon–Wiener diversity; (4) mean tolerance score (taxa scored from 1-10 with lower values indicating pollution tolerant taxa); and (5) the number of sensitive taxa (number of taxa with tolerance score >5 not including EPT).	(*86*)	Richness
Bulgaria	Biotic Index	Taxa are scored from 1-5 (lower values for taxa indicative of polluted conditions). The final metric value is based on the relative abundance of taxa with scores 1/2 versus 3/4/5.	(*87*)	NA
Cyprus	STAR Intercalibration Common Metric Index	(1) ASPT index; (2) Selected EPTD taxa (Ephemeroptera, Plecoptera, Trichoptera, Diptera); (3) 1-GOLD (% Gastropoda + Oligochaeta + Diptera); (4) Total family richness; (5) EPT family richness; (6) Shannon diversity	(*88*)	Richness, taxonomic composition

Czech Republic	Multimetric index using river type-specific metrics	Included metrics depend on river type. Can include: (1) % + total number of EPT; (2) total family richness; (3) Margalef diversity; (4) Czech saprobic index; (5) lithal microhabitat preference; (6) epirhithral, metarhithral, or hyporhithral, and/or epipotamal stream zone preferences; (7) % of different feeding types; (8) SPEAR organic sensitivity index; and (9) reconstructed taxocenoses (REKO) index; (10) Chironomidae taxon richness; (11) ratio of observed and and expected macroinvertebrate composition.	(*89*, *90*)	Abundance, richness, taxonomic and trait composition
Denmark	Danish Streamfauna Index	(1) The presence of six indicator taxa groups categorised by their resistance or sensitivity to organic pollution.	(*91*)	NA
England	Whalley Hawkes Paisley Trigg (WHPT) Average Score Per Taxon and number of scored taxa	Families are scored from -1.3–13 based on their known sensitivities to oxygen depletion, with higher scores for more sensitive families. $ASPT_{WHPT}$ is the sum of these scores divided by the number of scoring taxa.	(*92*, *93*)	NA
Estonia	Multimetric index using five metrics	(1) Total taxon richness; (2) Number of EPT (Ephemeroptera, Plecoptera and Trichoptera); (3) Shannon–Wiener diversity; (4) ASPT index	(*94*)	Richness, taxonomic composition

Finland	Finnish Multimetric Index	(1) Occurrence of type-specific reference taxa; (2) occurrence of type-specific reference EPT families; and (3) Percent Model Affinity (similarity of community composition to type-specific reference composition).	(*95–97*)	Taxonomic composition
France	I2M2	(1) Total taxon richness; (2) Shannon-Wiener diversity; (3) relative abundance of polyvoltine species; (4) relative abundance of ovoviviparous species; and (5) the Average Score Per Taxon index (ASPT; average score among taxa scored from 1-10, with lower values indicating taxa with greater pollution tolerance).	(*98*)	Richness, trait composition
Germany	Multimetric index using river type-specific metrics	Included metrics depend on river type. Can include: (1) the German Fauna Index (taxa assigned scores of -2 to +2, with lower values for taxa indicative of polluted conditions); (2) the Potamon Typie Index (affinity to the potamal zone); (3) the Rhithron Typie Index (affinity to the rhithral region); (4) the Lake Outlet Index (score 1-5 with higher values indicating taxa that prefer lake outlets); (3) % epirhithral taxa; (4) % metarhithral taxa; (5) % epipotamal taxa; (6) % littoral taxa; (7) % pelal taxa; (8) % phytal taxa; (9) ratio of rheophilic and rheobiont taxa; (10) total number of EPT; (11) total number of EPTCOB (EPT plus Coleoptera, Odonata, and Bivalvia); (12) % EPT; (13) % Oligochaeta.	(*99*)	Richness, taxonomic composition

Greece	Hellenic Evaluation System 2 (HESY2) and STAR_ICMi index	HESY2 is constructed using HES, a weighted taxon sensitivity score, and ASPT. STAR_ICMi index uses (1) ASPT index; (2) Selected EPTD taxa (Ephemeroptera, Plecoptera, Trichoptera, Diptera); (3) 1-GOLD (% Gastropoda + Oligochaeta + Diptera); (4) Total family richness; (5) EPT family richness; (6) Shannon diversity	(*100*, *101*)	Richness, taxonomic composition
Hungary	Hungarian Multimetric Index	Included metrics depend on river type. Always includes the ASPT index. Can include: (1) total number of EPT; (2) ratio of EPT; (3) total number of EPTCOB; (4) Shannon–Wiener diversity; (5) ratio of crenal to hyporhithral stream zonation preferences; (6) ratio of rheophil to rheobiont current preferences; (7) ratio of limno-to-rheophil to rheobiont current preferences.	(*102*)	Richness, Taxonomic composition
Ireland	Quality Rating System	Scoring taxa are pre-assigned into one of 5 groups from A–E, indicative of their sensitivity or tolerance to pollution (A being the most sensitive, E being the most tolerant). The final metric value is based on the categorical relative abundance of taxa within each group.	(*103*)	NA
Latvia	Latvian Macroinvertebrate Index	(1) Total taxon richness; (2) Shannon-Wiener diversity; (3) number of EPT; (4) Danish Streamfauna Index (DSFI; presence of six indicator taxa groups categorised by their resistance or sensitivity to organic pollution); and (5) the ASPT index.	(*104*)	Richness

Lithuania	Lithuanian River Macroinvertebrate Index	(1) Richness of Diptera, Ephemeroptera, and Plecoptera; (2) DSFI; (3) the ASPT index; and (4) (% Ephemeroptera + % Hemiptera + % Plecoptera) - (% Crustacea + % Hirudinea)	(*105*)	Richness, taxonomic composition
Luxembourg	I_2M_2	(1) Total taxon richness; (2) Shannon-Wiener diversity; (3) relative abundance of polyvoltine species; (4) relative abundance of ovoviviparous species; and (5) the Average Score Per Taxon index (ASPT; average score among taxa scored from 1-10, with lower values indicating taxa with greater pollution tolerance).	(*98*)	Richness, trait composition
Netherlands	KRW-maatlatten	(1) The abundance of positive and negative water type specific indicator taxa.	(*106*)	Abundance
Norway	Poorest out of the Average Score Per Taxon index and the River Acidification Macroinvertebrate Index	(1) ASPT index; (2) River Acidification Macroinvertebrate Index, which uses: (i) number of EPT and (ii) taxa scores based on tolerance to low pH.	(*95*)	Richness
Portugal	South Portugal macroinvertebrate biotic index	(1) Total number of families; (2) number of EPT; (3) the ASPT index (derived from Iberian BMWP scores); (4) total abundance of Chloroperlidae, Nemouridae, Leptophlebiidae, Ephemerellidae, Philipotamidae, Elmidae, Leuctridae, Limnephilidae, Sericostomatidae, Dryopidae and Athericidae.	(*107*)	Richness, abundance

Spain (Basque Country)	Multimetric Basque index family level (MBf index)	MBf index: (1) Total number of families; (2) number of EPT families; (3) abundance of a selection of 29 families of Ephemeroptera, Trichoptera, and Diptera; (4) abundance of a selection of 14 families of Ephemeroptera, Plecoptera, Trichoptera, and Diptera; (5) number of families from a selection of 12 families of Ephemeroptera, Trichoptera, and Diptera; and (6) IBMWP (described below) adapted to Basque rivers.	(*108–110*)	Richness, abundance
Spain (elsewhere)	Iberian Biological Monitoring Working Party (IBMWP)	IBMWP: Sum of scores across present families scored from 1–10 based on their known sensitivities to organic pollution, with higher scores for more sensitive families.	(*108–110*)	NA
Sweden	Average Score Per Taxon and the DJ index	(1) The ASPT index; and (2) the DJ index, which uses: (i) number of EPT, (ii) % EPT, (iii) and % Crustacea.	(*95*, *111*, *112*)	Richness, taxonomic composition
Switzerland	Multimetric index following the German system for a type 3.2 river	(1) Fauna index, a community composition index based on taxa-specific scores; (2) Rheoindex; (3) number of EPTCBO (Ephemeroptera, Plecoptera, Trichoptera, Coleoptera, Bivalvia, Odonata); (4) % of EPT	(*99*)	Richness, taxonomic composition

Methods and metrics used to produce the Ecological Quality Ratios (EQRs) for each country. The column ‘Circularity’ lists the metrics analyzed in this study that may introduce circularity because they are partly included in the EQR calculation.

Table S3.

Country	# of sites	Initial year	Final year	Mean sampling years	Mean time series duration	Sampling method(s)	Abundance units
Austria	1	2008	2019	8	12	Multi-habitat kick net (0.5mm mesh)	Ind. per m^2
Belgium	221	1989	2023	10.5	25.3	Multi-habitat kick net (0.3–0.5mm mesh) or colonization of artificial substrates	Ind. per sample
Bulgaria	8	1979	2019	8.4	17.6	Multi-habitat kick net (0.5mm mesh)	Ind. per sample
Cyprus	1	2008	2019	7	12	Multi-habitat kick net (0.5mm mesh)	Ind. per m^2
Czechia	129	2000	2021	9.1	14.8	Multi-habitat kick net (0.5mm mesh)	Ind. per sample
Denmark	245	1992	2020	19.7	22.4	Multi-habitat kick net (0.5mm mesh)	Ind. per 0.6 m^2
England	1,634	2002	2024	11.6	17.5	Multi-habitat kick net (1-mm mesh)	Ind. per sample
Estonia	2	2003	2019	9.5	15.5	Multi-habitat kick net (0.5mm mesh)	Ind. per 1.25 m^2
Finland	68	1998	2021	10.0	15.2	Multi-habitat kick net (0.5mm mesh)	Ind. per sample
France	918	1992	2022	9.4	14.3	Multi-habitat Surber sampler (0.5mm mesh)	Ind. per 0.4 m^2

Germany	110	1971	2021	9.5	16.7	Multi-habitat kick net (0.5mm mesh)	Ind. per 1.25 m^2
Greece	93	2012	2023	8.6	11.4	Multi-habitat kick net (0.5mm mesh)	Ind. per sample
Hungary	83	2005	2019	10.8	12.1	Multi-habitat kick net (0.5mm mesh)	Ind. per m^2
Ireland	116	2003	2023	8.4	15.3	Multi-habitat kick net (1-mm mesh)	Ind. per sample
Latvia	3	1986	2015	20.7	23.3	Multi-habitat kick net (0.5mm mesh)	Ind. per m^2
Lithuania	41	2010	2020	8.2	11.0	Multi-habitat kick net (0.5mm mesh)	Ind. per sample
Luxembourg	20	2007	2017	10.0	11.0	Multi-habitat Surber sampler (0.5mm mesh)	Ind. per sample
Netherlands	53	1980	2022	14.8	25.9	Multi-habitat kick net (0.5-mm mesh)	Ind. per 1.25 m^2
Norway	52	1981	2020	11.1	17.9	Multi-habitat kick net (0.5-mm mesh)	Ind. per sample
Portugal	2	1993	2019	27	27	Multi-habitat kick net (0.5mm mesh)	Ind. per sample
Spain	205	1986	2021	17.7	19.6	Multi-habitat kick net (0.5-mm mesh)	Ind. per 5 subsamples
Sweden	56	1995	2019	14.8	15.4	Multi-habitat kick net (0.5-mm mesh)	Ind. per m^2

Switzerland	1	1995	2018	8	24	Multi-habitat kick net (0.5mm mesh)	Ind. per sample
All	4,062	1971	2024	11.5	17.1	NA	NA

Descriptors of time series datasets by country.

Table S4.

Trait	Values	Modalities	Source	Coverage	
				No gap filling	Gap filled
Body size	0–3	<=0.25, >0.25–0.5, >0.5–1, >1–2, >2–4, >4–8, >8 cm	(*81*)	32%	86%
Dispersal mode	0–3	Aquatic passive, aquatic active, aerial passive, aerial active	(*81*)	32%	86%
Feeding type	0–10	Grazers/scrapers, miners, xylophagous, shredders, gatherers/collectors, active filter feeders, passive filter feeders, predators, parasites, other	(*80*)	88%	97%
Life duration	0–3	<1 year, >1 year	(*81*)	32%	86%
Locomotion	0–10	Swimming/skating, swimming/diving, burrowing/boring, sprawling/walking, sessile, other	(*80*)	78%	96%
Reproduction	0–3	Ovoviviparity, isolated eggs (free), isolated eggs (cemented), clutches (cemented or fixed), clutches (free), clutches (vegetation), clutches (terrestrial), asexual	(*81*)	27%	84%
Reproductive cycles per year	0–3	<1, 1, >1	(*81*)	32%	86%
Resistance forms	0–3	Eggs/gemmule/statoblasts, cocoons, housings against desiccation, diapause/dormancy, none	(*81*)	26%	83%
Respiration	0–3	Tegument, gill, plastron, spiracle, hydrostatic vesicle	(*81*)	27%	85%

Descriptions and sources for nine biological traits selected based on those commonly used in river invertebrate research (*113–116*). ‘Coverage’ lists the percent of taxa with available trait values with no gap filling and the percent with trait values after gap filling.

REFERENCES

1. Convention on Biological Diversity (CBD), "The Kunming-Montreal Global Biodiversity Framework" (CBD/COP/15/L25, 2022); www.cbd.int/doc/decisions/cop-15/cop-15-dec-04-en.pdf.

2. T. Newbold, L. N. Hudson, S. L. L. Hill, S. Contu, I. Lysenko, R. A. Senior, L. Börger, D. J. Bennett, A. Choimes, B. Collen, J. Day, A. De Palma, S. Díaz, S. Echeverria-Londoño, M. J. Edgar, A. Feldman, M. Garon, M. L. K. Harrison, T. Alhusseini, D. J. Ingram, Y. Itescu, J. Kattge, V. Kemp, L. Kirkpatrick, M. Kleyer, D. L. P. Correia, C. D. Martin, S. Meiri, M. Novosolov, Y. Pan, H. R. P. Phillips, D. W. Purves, A. Robinson, J. Simpson, S. L. Tuck, E. Weiher, H. J. White, R. M. Ewers, G. M. Mace, J. P. W. Scharlemann, A. Purvis, Global effects of land use on local terrestrial biodiversity. *Nature* **520**, 45–50 (2015).

3. F. Keck, T. Peller, R. Alther, C. Barouillet, R. Blackman, E. Capo, T. Chonova, M. Couton, L. Fehlinger, D. Kirschner, M. Knüsel, L. Muneret, R. Oester, K. Tapolczai, H. Zhang, F. Altermatt, The global human impact on biodiversity. *Nature* **641**, 395–400 (2025).

4. P. F. Langhammer, J. W. Bull, J. E. Bicknell, J. L. Oakley, M. H. Brown, M. W. Bruford, S. H. M. Butchart, J. A. Carr, D. Church, R. Cooney, S. Cutajar, W. Foden, M. N. Foster, C. Gascon, J. Geldmann, P. Genovesi, M. Hoffmann, J. Howard-McCombe, T. Lewis, N. B. W. Macfarlane, Z. E. Melvin, R. S. Merizalde, M. G. Morehouse, S. Pagad, B. Polidoro, W. Sechrest, G. Segelbacher, K. G. Smith, J. Steadman, K. Strongin, J. Williams, S. Woodley, T. M. Brooks, The positive impact of conservation action. *Science* **384**, 453–458 (2024).

5. P. Haase, D. Cortés-Guzmán, F. He, J. F. Jupke, T. Mangadze, F. M. Pelicice, M. A. Palmer, R. J. Rolls, R. B. Schäfer, E. A. R. Welti, J. S. Sinclair, Successes and failures of conservation actions to halt global river biodiversity loss. *Nat. Rev. Biodivers.* **1**, 104–118 (2025).

6. J. A. Catford, J. R. U. Wilson, P. Pyšek, P. E. Hulme, R. P. Duncan, Addressing context dependence in ecology. *Trends Ecol. Evol.* **37**, 158–170 (2022).

7. L. G. Shoemaker, L. L. Sullivan, I. Donohue, J. S. Cabral, R. J. Williams, M. M. Mayfield, J. M. Chase, C. Chu, W. S. Harpole, A. Huth, J. HilleRisLambers, A. R. M. James, N. J. B. Kraft, F. May, R. Muthukrishnan, S. Satterlee, F. Taubert, X. Wang, T. Wiegand, Q. Yang, K. C.

Abbott, Integrating the underlying structure of stochasticity into community ecology. *Ecology* **101**, e02922 (2020).

8. A. S. MacDougall, E. Harvey, J. L. McCune, K. A. Nilsson, J. Bennett, J. Firn, T. Bartley, J. B. Grace, J. Kelly, T. D. Tunney, B. McMeans, S.-I. S. Matsuzaki, T. Kadoya, E. Esch, K. Cazelles, N. Lester, K. S. McCann, Context-dependent interactions and the regulation of species richness in freshwater fish. *Nat. Commun.* **9**, 973 (2018).

9. L. S. Bittleston, M. Gralka, G. E. Leventhal, I. Mizrahi, O. X. Cordero, Context-dependent dynamics lead to the assembly of functionally distinct microbial communities. *Nat. Commun.* **11**, 1440 (2020).

10. P. Savary, J.-P. Lessard, P. R. Peres-Neto, Heterogeneous dispersal networks to improve biodiversity science. *Trends Ecol. Evol.* **39**, 229–238 (2024).

11. M. Vellend, L. Baeten, I. H. Myers-Smith, S. C. Elmendorf, R. Beauséjour, C. D. Brown, P. De Frenne, K. Verheyen, S. Wipf, Global meta-analysis reveals no net change in local-scale plant biodiversity over time. *Proc. Natl. Acad. Sci. U.S.A.* **110**, 19456–19459 (2013).

12. M. Dornelas, N. J. Gotelli, B. McGill, H. Shimadzu, F. Moyes, C. Sievers, A. E. Magurran, Assemblage time series reveal biodiversity change but not systematic loss. *Science* **344**, 296–299 (2014).

13. S. A. Blowes, S. R. Supp, L. H. Antão, A. Bates, H. Bruelheide, J. M. Chase, F. Moyes, A. Magurran, B. McGill, I. H. Myers-Smith, M. Winter, A. D. Bjorkman, D. E. Bowler, J. E. K. Byrnes, A. Gonzalez, J. Hines, F. Isbell, H. P. Jones, L. M. Navarro, P. L. Thompson, M. Vellend, C. Waldock, M. Dornelas, The geography of biodiversity change in marine and terrestrial assemblages. *Science* **366**, 339–345 (2019).

14. K. E. A. Norman, P. de Valpine, C. Boettiger, No general trend in functional diversity in bird and mammal communities despite compositional change. *Glob. Ecol. Biogeogr.* **34**, e13950 (2025).

15. A. Gonzalez, B. J. Cardinale, G. R. H. Allington, J. Byrnes, K. Arthur Endsley, D. G. Brown, D. U. Hooper, F. Isbell, M. I. O'Connor, M. Loreau, Estimating local biodiversity change: A critique of papers claiming no net loss of local diversity. *Ecology* **97**, 1949–1960 (2016).

16. B. J. Cardinale, A. Gonzalez, G. R. H. Allington, M. Loreau, Is local biodiversity declining or not? A summary of the debate over analysis of species richness time trends. *Biol. Conserv.* **219**, 175–183 (2018).

17. I. R. Staude, A. Weigelt, C. Wirth, Biodiversity change in light of succession theory. *Oikos* **2023**, e09883 (2023).

18. J. J. O'Grady, D. H. Reed, B. W. Brook, R. Frankham, What are the best correlates of predicted extinction risk? *Biol. Conserv.* **118**, 513–520 (2004).

19. L. Sykes, L. Santini, A. Etard, T. Newbold, Effects of rarity form on species' responses to land use. *Conserv. Biol.* **34**, 688–696 (2020).

20. A. Dellavalle, A. J. M. Devenish, C. Jarrett, N. N. D. Annorbah, A. Asamoah, K. Boafo, P. E. Lane, J. Owen, A. C. Morel, M. F. Hulme, A. J. Welch, K. Norris, F. J. Sanderson, J. A. Tobias, Shifting baselines increase the risk of misinterpreting biodiversity trends. *Ecography* **2026**, e08363 (2026).

21. V. Devictor, A. Robert, Measuring community responses to large-scale disturbance in conservation biogeography. *Divers. Distrib.* **15**, 122–130 (2009).

22. R. Elahi, M. I. O'Connor, J. E. K. Byrnes, J. Dunic, B. K. Eriksson, M. J. S. Hensel, P. J. Kearns, Recent trends in local-scale marine biodiversity reflect community structure and human impacts. *Curr. Biol.* **25**, 1938–1943 (2015).

23. C. Folke, S. Carpenter, B. Walker, M. Scheffer, T. Elmqvist, L. Gunderson, C. S. Holling, Regime shifts, resilience, and biodiversity in ecosystem management. *Annu. Rev. Ecol. Evol. Syst.* **35**, 557–581 (2004).

24. D. B. Lindenmayer, T. Lavery, B. C. Scheele, Why we need to invest in large-scale, long-term monitoring programs in landscape ecology and conservation biology. *Curr. Landsc. Ecol. Rep.* **7**, 137–146 (2022).

25. N. Bonada, N. Prat, V. H. Resh, B. Statzner, Developments in aquatic insect biomonitoring: A comparative analysis of recent approaches. *Annu. Rev. Entomol.* **51**, 495–523 (2006).

26. Water Framework Directive (WFD), Directive 2000/60/EC of the European Parliament and of the Council of 23 October 2000 establishing a framework for community action in the field of water policy. *Official Journal of the European Communities L* **327**, 1–72 (2000).

27. C. Bennett, R. Owen, S. Birk, A. Buffagni, S. Erba, N. Mengin, J. Murray-Bligh, G. Ofenböck, I. Pardo, W. van de Bund, F. Wagner, J.-G. Wasson, Bringing European river quality into line: An exercise to intercalibrate macro-invertebrate classification methods. *Hydrobiologia* **667**, 31–48 (2011).

28. R. van Klink, D. E. Bowler, K. B. Gongalsky, A. B. Swengel, A. Gentile, J. M. Chase, Meta-analysis reveals declines in terrestrial but increases in freshwater insect abundances. *Science* **368**, 417–420 (2020).

29. S. H. Hurlbert, The nonconcept of species diversity: A critique and alternative parameters. *Ecology* **52**, 577–586 (1971).

30. C.-H. Chiu, A. Chao, Distance-based functional diversity measures and their decomposition: A framework based on Hill numbers. *PLOS ONE* **9**, e100014 (2014).

31. M. O. Hill, H. G. Gauch, Detrended correspondence analysis: An improved ordination technique. *Vegetatio* **42**, 47–58 (1980).

32. S. Birk, W. Bonne, A. Borja, S. Brucet, A. Courrat, S. Poikane, A. Solimini, W. van de Bund, N. Zampoukas, D. Hering, Three hundred ways to assess Europe's surface waters: An almost complete overview of biological methods to implement the Water Framework Directive. *Ecol. Indic.* **18**, 31–41 (2012).

33. Y. Cao, A. W. Bark, W. P. Williams, Measuring the responses of macroinvertebrate communities to water pollution: A comparison of multivariate approaches, biotic and diversity indices. *Hydrobiologia* **341**, 1–19 (1996).

34. R. Thorne, P. Williams, The response of benthic macroinvertebrates to pollution in developing countries: A multimetric system of bioassessment. *Freshw. Biol.* **37**, 671–686 (1997).

35. T. E. L. Langford, P. J. Shaw, A. J. D. Ferguson, S. R. Howard, Long-term recovery of macroinvertebrate biota in grossly polluted streams: Re-colonisation as a constraint to ecological quality. *Ecol. Indic.* **9**, 1064–1077 (2009).

36. L. Mack, C. F. de la Hoz, M. Penk, J. Piggott, T. Crowe, D. Hering, W. Kaijser, J. Aroviita, J. Baer, A. Borja, D. E. Clark, Y. Fernández-Torquemada, J. Kotta, C. D. Matthaei, F. O'Beirn, H. W. Paerl, A. Sokolowski, A. Vilmi, S. Birk, Perceived multiple stressor effects depend on sample size and stressor gradient length. *Water Res.* **226**, 119260 (2022).

37. P. Segurado, C. Gutiérrez-Cánovas, T. Ferreira, P. Branco, Stressor gradient coverage affects interaction identification. *Ecol. Model.* **472**, 110089 (2022).

38. R. van Klink, D. E. Bowler, K. B. Gongalsky, M. Shen, S. R. Swengel, J. M. Chase, Disproportionate declines of formerly abundant species underlie insect loss. *Nature* **628**, 359–364 (2024).

39. K. L. Thompson, J. M. Chase, R. Remelgado, C. Meyer, The interacting effects of climate and land-cover change on bird communities in the United States. *Ecography* **2025**, e07949 (2025).

40. B. J. Kefford, R. Marchant, R. B. Schäfer, L. Metzeling, J. E. Dunlop, S. C. Choy, P. Goonan, The definition of species richness used by species sensitivity distributions approximates observed effects of salinity on stream macroinvertebrates. *Environ. Pollut.* **159**, 302–310 (2011).

41. S. A. Ludsin, M. W. Kershner, K. A. Blocksom, R. L. Knight, R. A. Stein, Life after death in Lake Erie: Nutrient controls drive fish species richness, rehabilitation. *Ecol. Appl.* **11**, 731–746 (2001).

42. T. H. Oliver, N. J. B. Isaac, T. A. August, B. A. Woodcock, D. B. Roy, J. M. Bullock, Declining resilience of ecosystem functions under biodiversity loss. *Nat. Commun.* **6**, 10122 (2015).

43. T. L. Weeks, P. A. Walkden, D. P. Edwards, A. C. Lees, A. L. Pigot, A. Purvis, J. A. Tobias, Land-use change undermines the stability of avian functional diversity. *Nature* **649**, 381–387 (2026).

44. European Parliament and Council of the European Union (EPCEU), *Regulation (EU) 2024/1991 of the European Parliament and of the Council of 24 June 2024 on Nature Restoration and Amending Regulation (EU) 2022/869* (2024); http://data.europa.eu/eli/reg/2024/1991/oj.

45. H. P. Jones, O. J. Schmitz, Rapid recovery of damaged ecosystems. *PLOS ONE* **4**, e5653 (2009).

46. P. Haase, D. E. Bowler, N. J. Baker, N. Bonada, S. Domisch, J. R. Garcia Marquez, J. Heino, D. Hering, S. C. Jähnig, A. Schmidt-Kloiber, R. Stubbington, F. Altermatt, M. Álvarez-Cabria, G. Amatulli, D. G. Angeler, G. Archambaud-Suard, I. A. Jorrín, T. Aspin, I. Azpiroz, I. Bañares, J. B. Ortiz, C. L. Bodin, L. Bonacina, R. Bottarin, M. Cañedo-Argüelles, Z. Csabai, T. Datry, E. de Eyto, A. Dohet, G. Dörflinger, E. Drohan, K. A. Eikland, J. England, T. E. Eriksen, V. Evtimova, M. J. Feio, M. Ferréol, M. Floury, M. Forcellini, M. A. E. Forio, R. Fornaroli, N. Friberg, J.-F. Fruget, G. Georgieva, P. Goethals, M. A. S. Graça, W. Graf, A. House, K.-L. Huttunen, T. C. Jensen, R. K. Johnson, J. I. Jones, J. Kiesel, L. Kuglerová, A. Larrañaga, P. Leitner, L. L'Hoste, M.-H. Lizée, A. W. Lorenz, A. Maire, J. A. M. Arnaiz, B. G. McKie, A. Millán, D. Monteith, T. Muotka, J. F. Murphy, D. Ozolins, R. Paavola, P. Paril, F. J. Peñas, F. Pilotto, M. Polášek, J. J. Rasmussen, M. Rubio, D. Sánchez-Fernández, L. Sandin, R. B. Schäfer, A. Scotti, L. Q. Shen, A. Skuja, S. Stoll, M. Straka, H. Timm, V. G. Tyufekchieva, I. Tziortzis, Y. Uzunov, G. H. van der Lee, R. Vannevel, E. Varadinova, G. Várbíró, G. Velle, P. F. M. Verdonschot, R. C. M. Verdonschot, Y. Vidinova, P. Wiberg-

Larsen, E. A. R. Welti, The recovery of European freshwater biodiversity has come to a halt. *Nature* **620**, 582–588 (2023).

47. J. S. Sinclair, E. A. R. Welti, F. Altermatt, M. Álvarez-Cabria, J. Aroviita, N. J. Baker, L. Barešová, J. Barquín, L. Bonacina, N. Bonada, M. Cañedo-Argüelles, Z. Csabai, E. de Eyto, A. Dohet, G. Dörflinger, T. E. Eriksen, V. Evtimova, M. J. Feio, M. Ferréol, M. Floury, M. A. E. Forio, R. Fornaroli, P. L. M. Goethals, J. Heino, D. Hering, K.-L. Huttunen, S. C. Jähnig, R. K. Johnson, L. Kuglerová, B. Kupilas, L. L'Hoste, A. Larrañaga, P. Leitner, A. W. Lorenz, B. G. McKie, T. Muotka, D. Osadčaja, R. Paavola, V. Palinauskas, P. Pařil, F. Pilotto, M. Polášek, J. J. Rasmussen, R. B. Schäfer, A. Schmidt-Kloiber, A. Scotti, A. Skuja, M. Straka, R. Stubbington, H. Timm, V. Tyufekchieva, I. Tziortzis, R. Vannevel, G. Várbíró, G. Velle, R. C. M. Verdonschot, S. Vray, P. Haase, Multi-decadal improvements in the ecological quality of European rivers are not consistently reflected in biodiversity metrics. *Nat. Ecol. Evol.* **8**, 430–441 (2024).

48. A. Lyche Solheim, J.-E. Thrane, S. Mentzel, S. J. Moe, Harmonised biological indicators for rivers and lakes: Towards European assessment of temporal trends in ecological quality. *Ecol. Indic.* **171**, 113207 (2025).

49. M. Vos, D. Hering, M. O. Gessner, F. Leese, R. B. Schäfer, R. Tollrian, J. Boenigk, P. Haase, R. Meckenstock, D. Baikova, H. Bayat, A. Beermann, D. Beisser, B. Beszteri, S. Birk, L. Boden, V. Brauer, M. Brauns, D. Buchner, A. Burfeid-Castellanos, G. David, A. Deep, A. Doliwa, M. Dunthorn, J. Enß, C. Escobar-Sierra, C. K. Feld, N. Fohrer, D. Grabner, U. Hadziomerovic, S. C. Jähnig, M. Jochmann, S. Khaliq, J. Kiesel, A. Kuppels, K. P. Lampert, T. T. Y. Le, A. W. Lorenz, G. M. Madariaga, B. Meyer, J. H. Pantel, I. M. Pimentel, N. S. Mayombo, H. H. Nguyen, K. Peters, S. M. Pfeifer, S. Prati, A. J. Probst, D. Reiner, P. Rolauffs, A. Schlenker, T. C. Schmidt, M. Shah, G. Sieber, T. L. Stach, A.-K. Tielke, A.-M. Vermiert, M. Weiss, M. Weitere, B. Sures, The Asymmetric Response Concept explains ecological consequences of multiple stressor exposure and release. *Sci. Total Environ.* **872**, 162196 (2023).

50. H. P. Jones, P. C. Jones, E. B. Barbier, R. C. Blackburn, J. M. R. Benayas, K. D. Holl, M. McCrackin, P. Meli, D. Montoya, D. M. Mateos, Restoration and repair of Earth's damaged ecosystems. *Proc. R. Soc. B Biol. Sci.* **285**, 20172577 (2018).

51. R. J. Fletcher Jr., R. E. Green, E. K. Bladon, P. W. Atkinson, B. T. Phalan, D. Williams, P. Visconti, A. Balmford, Beyond species richness for biological conservation. *Conserv. Lett.* **18**, e13124 (2025).

52. J. S. Sinclair, R. Stubbington, R. B. Schäfer, L. Barešová, N. Bonada, Z. Csabai, J. I. Jones, A. Larrañaga, J. F. Murphy, P. Pařil, M. Polášek, J. J. Rasmussen, M. Straka, G. Várbíró, R. C. M. Verdonschot, E. A. R. Welti, P. Haase, Ecological but not biological traits of European riverine invertebrates respond consistently to anthropogenic impacts. *Glob. Ecol. Biogeogr.* **33**, e13931 (2024).

53. H. Hillebrand, B. Blasius, E. T. Borer, J. M. Chase, J. A. Downing, B. K. Eriksson, C. T. Filstrup, W. S. Harpole, D. Hodapp, S. Larsen, A. M. Lewandowska, E. W. Seabloom, D. B. Van de Waal, A. B. Ryabov, Biodiversity change is uncoupled from species richness trends: Consequences for conservation and monitoring. *J. Appl. Ecol.* **55**, 169–184 (2018).

54. M. Boënnec, V. Dakos, V. Devictor, Sources of confusion in global biodiversity trends. *Oikos* **2024**, e10732 (2024).

55. J. M. Chase, B. J. McGill, P. L. Thompson, L. H. Antão, A. E. Bates, S. A. Blowes, M. Dornelas, A. Gonzalez, A. E. Magurran, S. R. Supp, M. Winter, A. D. Bjorkman, H. Bruelheide, J. E. K. Byrnes, J. S. Cabral, R. Elahi, C. Gomez, H. M. Guzman, F. Isbell, I. H. Myers-Smith, H. P. Jones, J. Hines, M. Vellend, C. Waldock, M. O'Connor, Species richness change across spatial scales. *Oikos* **128**, 1079–1091 (2019).

56. M. Desquilbet, L. Gaume, M. Grippa, R. Céréghino, J.-F. Humbert, J.-M. Bonmatin, P.-A. Cornillon, D. Maes, H. Van Dyck, D. Goulson, Comment on "Meta-analysis reveals declines in terrestrial but increases in freshwater insect abundances". *Science* **370**, eabd8947 (2020).

57. J. W. Valdez, C. T. Callaghan, J. Junker, A. Purvis, S. L. L. Hill, H. M. Pereira, The undetectability of global biodiversity trends using local species richness. *Ecography* **2023**, e06604 (2023).

58. P. Haase, S. Lohse, S. Pauls, K. Schindehütte, A. Sundermann, P. Rolauffs, D. Hering, Assessing streams in Germany with benthic invertebrates: Development of a practical standardised protocol for macroinvertebrate sampling and sorting. *Limnologica* **34**, 349–365 (2004).

59. S. J. Moe, S. Mentzel, S. A. Welch, A. Lyche Solheim, From national monitoring to transnational indicators: Reporting and processing of aquatic biology data under the European Environment Agency's State of the Environment data flow. *Front. Environ. Sci.* **11**, 1057742 (2023).

60. M. Lumbierres, M. Milanović, P. Beja, A. Bonn, T. D. Breeze, L. Brotons, N. Fernández, J. Junker, C. Liquete, A. L. Solheim, A. M. Ordóñez, F. Moreira, J. Santana, S. Shinneman, B. Smets, H. M. Pereira, J. W. Valdez, R. H. A. van Grunsven, W. D. Kissling, Towards implementing workflows for essential biodiversity variables at a European scale. *Glob. Ecol. Conserv.* **62**, e03699 (2025).

61. M. Cañedo-Argüelles, M. Beklioğlu, J. Crabot, D. Cunillera-Montcusí, G. Free, D. Hernández-Carrasco, M. G. Kelly, A. J. Moyano Salcedo, R. B. Schäfer, S. Poikane, *Preparing European Freshwaters for a Saltier Future* (Publications Office of the European Union, 2025); https://data.europa.eu/doi/10.2760/7705629.

62. G. Free, S. Poikane, A. L. Solheim, M. Bussettini, C. Bradley, J. Smith, R. Caroni, M. Bresciani, M. Pinardi, C. Giardino, W. van de Bund, Climate change and ecological assessment in Europe under the WFD–Hitting moving targets with shifting baselines? *J. Environ. Manage.* **370**, 122884 (2024).

63. H. Hillebrand, J.-C. Dajka, M. Halbach, A. Happe, R. Röchert, R. Seppelt, J. Settele, M. Weitere, M. Winter, Y. Zinngrebe, D. Hodapp, Operational perspectives for biodiversity indicators. *Ecol. Solut. Evid.* **6**, e70134 (2025).

64. G. N. Saari, Z. Wang, B. W. Brooks, Revisiting inland hypoxia: Diverse exceedances of dissolved oxygen thresholds for freshwater aquatic life. *Environ. Sci. Pollut. Res.* **25**, 3139–3150 (2018).

65. M. J. McNellie, I. Oliver, J. Dorrough, S. Ferrier, G. Newell, P. Gibbons, Reference state and benchmark concepts for better biodiversity conservation in contemporary ecosystems. *Glob. Chang. Biol.* **26**, 6702–6714 (2020).

66. D. A. Kopp, J. L. Stoddard, P. R. Kaufmann, A. T. Herlihy, R. A. Hill, M. M. Brehob, R. D. Sabo, A model-based assessment of anthropogenic disturbance on lotic macroinvertebrate assemblages. *Ecosphere* **16**, e70361 (2025).

67. A. Ramos-Merchante, P. Sáez-Gómez, J. Prenda, Historical distribution of freshwater fishes and the reference conditions concept in a large Mediterranean basin. *Aquat. Conserv. Mar. Freshw. Ecosyst.* **31**, 888–902 (2021).

68. S. Birk, L. van Kouwen, N. Willby, Harmonising the bioassessment of large rivers in the absence of near-natural reference conditions – a case study of the Danube River. *Freshw. Biol.* **57**, 1716–1732 (2012).

69. I. Acosta-Alba, H. M. G. Van der Werf, The use of reference values in indicator-based methods for the environmental assessment of agricultural systems. *Sustainability* **3**, 424–442 (2011).

70. M. A. Wilkes, M. Mungee, M. Naura, V. A. Bell, L. E. Brown, Predicting nature recovery for river restoration planning and ecological assessment: A case study from England, 1991–2042. *River Res. Appl.* **41**, 68–81 (2025).

71. E. A. R. Welti, D. E. Bowler, J. S. Sinclair, F. Altermatt, M. Álvarez-Cabria, G. Amatulli, D. G. Angeler, G. Archambaud, I. Arrate Jorrín, T. Aspin, I. Azpiroz, N. J. Baker, I. Bañares, J. Barquín Ortiz, C. L. Bodin, L. Bonacina, N. Bonada, R. Bottarin, M. Cañedo-Argüelles, Z. Csabai, T. Datry, E. de Eyto, A. Dohet, S. Domisch, G. Dörflinger, E. Drohan, K. A. Eikland, J. England, T. E. Eriksen, V. Evtimova, M. J. Feio, M. Ferréol, M. Floury, M. Forcellini, M. A. E. Forio, R. Fornaroli, N. Friberg, J.-F. Fruget, J. R. Garcia Marquez, G. Georgieva, P.

Goethals, M. A. S. Graça, A. House, K.-L. Huttunen, T. C. Jensen, R. K. Johnson, J. I. Jones, J. Kiesel, A. Larrañaga, P. Leitner, L. L'Hoste, M.-H. Lizée, A. W. Lorenz, A. Maire, J. A. Manzanos Arnaiz, B. Mckie, A. Millán, T. Muotka, J. F. Murphy, D. Ozolins, R. Paavola, P. Paril, F. J. Peñas Silva, M. Polasek, J. Rasmussen, M. Rubio, D. Sánchez Fernández, L. Sandin, R. B. Schäfer, A. Schmidt-Kloiber, A. Scotti, L. Q. Shen, A. Skuja, S. Stoll, M. Straka, R. Stubbington, H. Timm, V. G. Tyufekchieva, I. Tziortzis, Y. Uzunov, G. H. van der Lee, R. Vannevel, E. Varadinova, G. Várbíró, G. Velle, P. F. M. Verdonschot, R. C. M. Verdonschot, Y. Vidinova, P. Wiberg-Larsen, P. Haase, Time series of freshwater macroinvertebrate abundances and site characteristics of European streams and rivers. *Sci. Data* **11**, 601 (2024).

72. J. Heino, J. Soininen, Are higher taxa adequate surrogates for species-level assemblage patterns and species richness in stream organisms? *Biol. Conserv.* **137**, 78–89 (2007).

73. S. N. Wood, *Generalized Additive Models: An Introduction with R* (Chapman and Hall/CRC, ed. 2, 2017).

74. R Core Team, R: A language and environment for statistical computing, version 4.5.1 (R Foundation for Statistical Computing, 2025); www.r-project.org/.

75. P. Legendre, E. D. Gallagher, Ecologically meaningful transformations for ordination of species data. *Oecologia* **129**, 271–280 (2001).

76. J. Oksanen, G. Simpson, F. Blanchet, R. Kindt, P. Legendre, P. Minchin, R. O'Hara, P. Solymos, M. Stevens, E. Szoecs, H. Wagner, M. Barbour, M. Bedward, B. Bolker, D. Borcard, T. Borman, G. Carvalho, M. Chirico, M. De Caceres, S. Durand, H. Evangelista, R. FitzJohn, M. Friendly, B. Furneaux, G. Hannigan, M. Hill, L. Lahti, C. Martino, D. McGlinn, M. Ouellette, E. Ribeiro Cunha, T. Smith, A. Stier, C. Ter Braak, J. Weedon, vegan: Community ecology package, version 2.7-2 (2025); https://CRAN.R-project.org/package=vegan.

77. T. C. Hsieh, K. H. Ma, A. Chao, iNEXT: An R package for rarefaction and extrapolation of species diversity (Hill numbers). *Methods Ecol. Evol.* **7**, 1451–1456 (2016).

78. S. Lavorel, K. Grigulis, S. McIntyre, N. S. G. Williams, D. Garden, J. Dorrough, S. Berman, F. Quétier, A. Thébault, A. Bonis, Assessing functional diversity in the field – methodology matters! *Funct. Ecol.* **22**, 134–147 (2008).

79. D. Li, hillR: Taxonomic, functional, and phylogenetic diversity and similarity through Hill Numbers. *J. Open Source Softw.* **3**, 1041 (2018).

80. A. Schmidt-Kloiber, D. Hering, www.freshwaterecology.info – An online tool that unifies, standardises and codifies more than 20,000 European freshwater organisms and their ecological preferences. *Ecol. Indic.* **53**, 271–282 (2015).

81. H. Tachet, P. Richoux, M. Bournaud, P. Usseglio-Polatera, *Invertébrés d'eau Douce: Systématique, Biologie, Écologie* (CNRS Éditions, 2010), vol. 15 [Freshwater invertebrates: Systematics, Biology, Ecology].

82. S. Kunz, B. J. Kefford, A. Schmidt-Kloiber, C. D. Matthaei, P. Usseglio-Polatera, W. Graf, N. L. Poff, L. Metzeling, L. Twardochleb, C. P. Hawkins, R. B. Schäfer, Tackling inconsistencies among freshwater invertebrate trait databases: Harmonising across continents and aggregating taxonomic resolution. *Freshw. Biol.* **67**, 275–291 (2022).

83. S. Lê, J. Josse, F. Husson, FactoMineR: An R package for multivariate analysis. *J. Stat. Softw.* **25**, 1–18 (2008).

84. S. Dray, R. Pélissier, P. Couteron, M.-J. Fortin, P. Legendre, P. R. Peres-Neto, E. Bellier, R. Bivand, F. G. Blanchet, M. De Cáceres, A.-B. Dufour, E. Heegaard, T. Jombart, F. Munoz, J. Oksanen, J. Thioulouse, H. H. Wagner, Community ecology in the age of multivariate multiscale spatial analysis. *Ecological monographs* **82**, 257–275 (2012).

85. T. Ofenböck, O. Moog, J. Gerritsen, M. Barbour, "A stressor specific multimetric approach for monitoring running waters in Austria using benthic macro-invertebrates" in *Integrated Assessment of Running Waters in Europe*, D. Hering, P. F. M. Verdonschot, O. Moog, L. Sandin, Eds. (Springer Netherlands, 2004), pp. 251–268; https://doi.org/10.1007/978-94-007-0993-5_15.

86. W. Gabriels, K. Lock, N. De Pauw, P. L. M. Goethals, Multimetric Macroinvertebrate Index Flanders (MMIF) for biological assessment of rivers and lakes in Flanders (Belgium). *Limnologica* **40**, 199–207 (2010).

87. S. Cheshmedjiev, E. Varadinova, "Bottom macroinvertebrates" in *Biological Analysis and Ecological Assessment of Surface Water Types in Bulgaria*, D. Belkinova, G. Gechev, S. Cheshmejhiev, I. Dimitrova-Dulgerova, R. Mladenov, M. Marinov, I. Tenev, P. Stoyanov, P. Ivanov, S. Mikhov, L. Pehlivanov, E. Varadinova, T. Karagyozova, M. Vasilev, A. Apostolu, B. Velkov, M. Pavlova, Eds. (P. Hilendarski Univ. Publishing House, 2013), pp. 147–162.

88. M. J. Feio, J. Ferreira, A. Buffagni, S. Erba, G. Dörflinger, M. Ferréol, A. Munné, N. Prat, I. Tziortzis, G. Urbanič, Comparability of ecological quality boundaries in the Mediterranean basin using freshwater benthic invertebrates. Statistical options and implications. *Sci. Total Environ.* **476-477**, 777–784 (2014).

89. T. Mičaník, P. Vyskoč, H. Prchalová, M. Polášek, D. Němejcová, M. Durčák, P. Richter, Hodnocení stavu útvarů povrchových vod v České republice pro 3. plánovací období plánů povodí. *Vodohospodářské technicko-ekonomické informace* **62**, 4–18 (2020) [Surface Water Status Assessment for the Third Cycle River basin Management Plan of the Czech Republic].

90. L. Opatřilová, D. Němejcová, S. Zahrádková, J. Kokeš, "Assessment method for ecological status of rivers based on biological quality element benthic invertebrates in the Czech Republic" (Karkonosze, Przesieka, 2015), p. 3.

91. J. Skriver, N. Friberg, J. Kirkegaard, Biological assessment of running waters in Denmark: Introduction of the Danish Stream Fauna Index (DSFI). *SIL Proc.* **27**, 1822–1830 (2000).

92. J. Murray-Bligh, M. Griffiths, *Freshwater Biology and Ecology Handbook* (Foundation for Water Research & Freshwater Biological Association, 2022); http://www.fwrinformationcentre.co.uk/biology-and-ecology-handbook/.

93. M. F. Paisley, D. J. Trigg, W. J. Walley, Revision of the Biological Monitoring Working Party (BMWP) score system: Derivation of present-only and abundance-related scores from field data. *River Res. Appl.* **30**, 887–904 (2014).

94. K. Käiro, T. Möls, H. Timm, T. Virro, R. Järvekülg, The effect of damming on biological quality according to macroinvertebrates in some Estonian streams, Central—Baltic Europe: A pilot study. *River Res. Appl.* **27**, 895–907 (2011).

95. J. H. Andersen, J. Aroviita, J. Carstensen, N. Friberg, R. K. Johnson, P. Kauppila, M. Lindegarth, C. Murray, K. Norling, Approaches for integrated assessment of ecological and eutrophication status of surface waters in Nordic Countries. *Ambio* **45**, 681–691 (2016).

96. J. Aroviita, E. Koskenniemi, J. Kotanen, H. Hämäläinen, A priori typology-based prediction of benthic macroinvertebrate fauna for ecological classification of rivers. *Environ. Manag.* **42**, 894–906 (2008).

97. J. Aroviita, S. Mitikka, S. Vienonen, "Status classification and assessment criteria of surface waters in the third river basin management cycle" (37/2019, Suomen ympäristökeskuksen raportteja, 2019); http://hdl.handle.net/10138/306745.

98. C. P. Mondy, B. Villeneuve, V. Archaimbault, P. Usseglio-Polatera, A new macroinvertebrate-based multimetric index (I2M2) to evaluate ecological quality of French wadeable streams fulfilling the WFD demands: A taxonomical and trait approach. *Ecol. Indic.* **18**, 452–467 (2012).

99. E. Berger, P. Haase, M. Kuemmerlen, M. Leps, R. B. Schäfer, A. Sundermann, Water quality variables and pollution sources shaping stream macroinvertebrate communities. *Sci. Total Environ.* **587-588**, 1–10 (2017).

100. M. Lazaridou, C. Ntislidou, I. Karaouzas, N. Skoulikidis, S. Birk, Harmonization of the assessment method for classifying the ecological quality status of very large Greek rivers. *Knowl. Manag. Aquat. Ecosyst.* **419**, 50 (2018).

101. M. Lazaridou, C. Ntislidou, I. Karaouzas, N. Skoulikidis, Harmonisation of a new assessment method for estimating the ecological quality status of Greek running waters. *Ecol. Indic.* **84**, 683–694 (2018).

102. T. Bozóki, E. Á. Krasznai-Kun, A. Csercsa, G. Várbíró, P. Boda, Temporal and spatial dynamics in aquatic macroinvertebrate communities along a small urban stream. *Environ. Earth Sci.* **77**, 559 (2018).

103. H. B. Feeley, C. Bradley, G. Free, B. Kennedy, R. Little, N. McDonnell, C. Plant, W. Trodd, C. Wynne, S. O. Boyle, A national macroinvertebrate dataset collected for the biomonitoring of Ireland's river network, 2007–2018. *Sci. Data* **7**, 280 (2020).

104. D. Ozoliņš, I. Karklina, A. Skuja, L. Uzule, I. Kokorite, M. Medne-Peipere, A. Lazdiņš, The impact of forest fertilization on the ecological quality of two hemiboreal streams. *Forests* **13**, 196 (2022).

105. E. Šidagytė-Copilas, K. Arbačiauskas, A multimetric macroinvertebrate index for the assessment of the ecological status of Lithuanian rivers. *Limnologica* **97**, 126010 (2022).

106. W. Altenburg, G. Arts, J. G. Baretta-Bekker, M. S. van den Berg, T. van den B. Broek, R. Buskens, R. Bijkerk, H. C. Coops, H. van Dam, G. van Ee, C. H. M. Evers, R. Franken, B. Higler, T. Ietswaart, N. Jaarsma, D. J. de Jong, A. M. T. Joosten, M. Klinge, R. a. E. Knoben, J. Kranenbarg, W. M. G. M. van Loon, R. Noordhuis, R. Pot, F. Twisk, P. F. M. Verdonschot, H. Vlek, J. J. G. M. Backx, M. Beers, A. D. Buijse, G. Duursema, M. Fagel, J. de Leeuw, J. van der Molen, R. C. Nijboer, J. Postma, T. Vriese, R. Duijts, J. G. Hartholt, Z. Jager, E. C. Stikvoort, D. Walvoort, "Referenties en maatlatten voor natuurlijke watertypen voor de Kaderrichtlijn Water 2021–2027" (2018–49, Stowa, 2018); https://edepot.wur.nl/469646 [References and Performance Standards for Natural Water Bodies under the Water Framework Directive 2021–2027].

107. F. Leitão, S. J. Hughes, I. Máximo, N. Atanasova, A. Furtado, L. Chicharo, Habitat-oriented sampling of macroinvertebrates affects the determination of ecological status in temporary mediterranean river systems. *River Res. Appl.* **30**, 1233–1247 (2014).

108. J. Alba-Tercedor, P. Jáimez-Cuéllar, M. Álvarez, J. Avilés, N. Bonada, J. Casas, A. Mellado, M. Ortega, I. Pardo, N. Prat, M. Rieradeva, S. Robles, C. E. Sáinz-Cantero, A. Sánchez-Ortega, M. L. Suárez, M. Toro, M. R. Vidal-Abarca, S. Vivas, C. Zamora-Muñoz, Caracterización del estado ecológico de ríos mediterráneos ibéricos mediante el índice

IBMWP (antes BMWP'). *Limnetica* **21**, 175–185 (2002) [Characterization of the ecological status of Iberian Mediterranean rivers using the IBMWP index (formerly BMWP)].

109. B. G. De Bikuña, E. López, J. M. Leonardo, J. Arrate, A. Martínez, A. Manzanos, Development of a multimetric benthic macroinvertebrate index for assessing the ecological condition of Basque streams (north of Spain). *Fundam. Appl. Limnol.* **187**, 21–32 (2015).

110. A. Munné, N. Prat, Use of macroinvertebrate-based multimetric indices for water quality evaluation in Spanish Mediterranean rivers: An intercalibration approach with the IBMWP index. *Hydrobiologia* **628**, 203–225 (2009).

111. M. A. Bighiu, S. Höss, W. Traunspurger, M. Kahlert, W. Goedkoop, Limited effects of pesticides on stream macroinvertebrates, biofilm nematodes, and algae in intensive agricultural landscapes in Sweden. *Water Res.* **174**, 115640 (2020).

112. J. Dahl, R. K. Johnson, A multimetric macroinvertebrate index for detecting organic pollution of streams in southern Sweden. *Archiv für Hydrobiologie* **160**, 487–513 (2004).

113. S. Dolédec, B. Statzner, M. Bournard, Species traits for future biomonitoring across ecoregions: Patterns along a human-impacted river. *Freshw. Biol.* **42**, 737–758 (1999).

114. S. Dolédec, B. Statzner, Invertebrate traits for the biomonitoring of large European rivers: An assessment of specific types of human impact. *Freshw. Biol.* **53**, 617–634 (2008).

115. C. P. Mondy, P. Usseglio-Polatera, Using fuzzy-coded traits to elucidate the non-random role of anthropogenic stress in the functional homogenisation of invertebrate assemblages. *Freshw. Biol.* **59**, 584–600 (2014).

116. E. Berger, P. Haase, R. B. Schäfer, A. Sundermann, Towards stressor-specific macroinvertebrate indices: Which traits and taxonomic groups are associated with vulnerable and tolerant taxa? *Sci. Total Environ.* **619-620**, 144–154 (2018).
